# Malting quality and preharvest sprouting traits are genetically correlated in spring malting barley

**DOI:** 10.1007/s00122-023-04257-6

**Published:** 2023-03-13

**Authors:** Travis E. Rooney, Daniel W. Sweeney, Karl H. Kunze, Mark E. Sorrells, Jason G. Walling

**Affiliations:** 1 Plant Breeding and Genetics Section, School of Integrative Plant Sciences, Cornell University, Ithaca, NY 14853 USA; 2 USDA–ARS - Cereal Crops Research Unit, 502 Walnut St, Madison, WI 53726 USA

## Abstract

**Key message:**

Malt for craft “all-malt” brewing can have high quality, PHS resistance, and malted in normal timeframes. Canadian style adjunct malt is associated with PHS susceptibility.

**Abstract:**

Expansion of malting barley production into non-traditional growing regions and erratic weather has increased the demand for preharvest sprouting (PHS) resistant, high quality malting barley cultivars. This is hindered by the relatively unknown relationships between PHS resistance and malting quality. Here we present a three-year study of malting quality and germination at different after-ripening durations post physiological maturity. Malting quality traits alpha amylase (AA) and free amino nitrogen (FAN) and germination rate at six days post PM shared a common association with a SNP in *HvMKK3* on chromosome 5H in the Seed Dormancy 2 (*SD2*) region responsible for PHS susceptibility. Soluble protein (SP) and soluble over total protein (S/T) both shared a common association with a marker in the *SD2* region. Significant genetic correlations between PHS resistance and the malting quality traits AA, FAN, SP, S/T were detected across and within *HvMKK3* allele groups. High adjunct malt quality was related to PHS susceptibility. Selection for PHS resistance led to a correlated response in malting quality traits. Results strongly suggest pleiotropy of *HvMKK3* on malting quality traits and that the classic “Canadian-style” malt is caused by a PHS susceptible allele of *HvMKK3*. PHS susceptibility appears to benefit the production of malt intended for adjunct brewing, while PHS resistance is compatible with all-malt brewing specifications. Here we present our analysis on the effect of combining complexly inherited and correlated traits with contrasting goals to inform breeding practice in malting barley, the general principles of which can be extended to other breeding programs.

**Supplementary Information:**

The online version contains supplementary material available at 10.1007/s00122-023-04257-6.

## Introduction

Spring malting barley (*Hordeum vulgare* L.) is grown worldwide for the production of malt, an ingredient in many alcoholic beverages and food products. Malting is a process of controlled germination where barley is steeped, germinated, and then dried (kilned) to develop flavor compounds and promote shelf stability (Briggs 1998). Preharvest sprouting (PHS) (visible sprouting) or pre-germination (non-visible sprouting) occur in cereals when mature spikes become wetted and begin to germinate in the field. Both PHS and pre-germinated barley fail to meet malt quality standards and are sold at a substantial economic loss to the grower. Preharvest sprouting resistance requires seed dormancy (Rodríguez et al. 2015; Ullrich et al. 2009), defined as the inability of a viable seed to germinate in favorable conditions (Bewley et al. 2013; Depauw and Mccaig 1991). Dormancy can lead to poor germination during malting which may affect the quality of malt produced. Expansion of barley production to novel areas and greater potential for erratic weather patterns (Herring et al. 2018) in historical production regions have increased the demand for PHS resistant, high malting quality barley varieties. The development of such varieties is hindered by a paucity of information on the relationships between PHS resistance and malting quality, the expense of phenotyping malting quality traits, and concerns over storage costs in malthouses.

Malt quality is assessed with multiple traits, both prior to malting and on the malted grain. For grain, kernel plumpness and protein content are commonly measured (*Malting Barley Breeding *Guidelines 2019). Malt enzymatic activity is measured with two traits, diastatic power (DP) and alpha amylase activity (AA), both direct measures of starch hydrolysis rates. Malt extract (ME) is the percentage of soluble material in the malt that is solubilized into the wort (the sugary liquid produced during mashing). Soluble protein (SP) is the amount of protein solubilized into the wort and free amino nitrogen (FAN) is the concentration of free amine groups in the wort. (Ullrich et al. 1997; Briggs 2002). Other common traits include soluble/total protein (S/T), the ratio of SP to total malt protein (MP) (S/T = SP/MP) and wort beta-glucan (BG) content. Each of these traits is predictive of how well a malt will perform when mashed and used in brewing or distilling.

Many studies have focused on the genetic mapping of malting quality traits (Beattie et al. 2010; Joe et al. 2016; Looseley et al. 2020; Mohammadi et al. 2015; Pauli et al. 2018). Others have focused on the genetic mapping of dormancy (Han et al. 1999; Hickey et al. 2012; Li et al. 2003; Lin et al. 2009; Prada et al. 2004; Sato et al. 2009; Sweeney et al. 2021a, b; Ullrich et al. 2009). These studies reported that there are loci on the long arm of chromosome 5H that are associated with malting quality and seed dormancy/PHS susceptibility. The nature of this association remains unclear, whether it be tightly linked genes or a major locus affecting all traits. This region has been called the Seed Dormancy 2 (*SD2*) locus in regard to the dormancy associations. Nakamura et al. (2016) demonstrated that mutations within a Mitogen Activated Kinase Kinase3 gene (*HvMKK3*) in the *SD2* region are responsible for changes in dormancy. Vetch et al. (2020) identified four alleles of this gene in North American germplasm and associated the E165Q mutation with the changes in dormancy and PHS susceptibility. Sweeney et al. (2021a, b) identified two additional alleles that were associated with changes in dormancy phenotypes over the “wild-type” (dormant) alleles, and presented evidence suggesting that one allele, the highly non-dormant *HvMKK3*, may have pleiotropic effects on malting quality traits. The *HvMKK3* alleles were designated as “highly non-dormant” (MKK3_N*_), “non-dormant” (MKK3_N_), and “dormant” (MKK3_D_) according to their phenotypes.

Additional evidence for a common association of malting quality and dormancy in the *SD2* region was provided in Castro et al. (2010) who found common quantitative trait loci for dormancy at maturity and malting quality traits on the end of chromosome 5H, in the *SD2* region. These associations they found were significant for FAN, AA, and S/T. Edney et al. (2013) examined a biparental population between a PHS susceptible Canadian breeding line with “Canadian-style” malting quality and PHS resistant Australian malting barley, Baudin. They employed a PCR based marker (GMS001) linked to the MKK3_N*_ allele of *HvMKK3* enriched in Canadian spring malting barley and either the MKK3_D_, MKK3_N_, or another unidentified allele of *HvMKK3* from the Australian germplasm pool. The screen identified lines that successfully combined the PHS resistance of Baudin with Canadian style malting quality, albeit on the lower end of the range for the FAN and AA traits.

Controlling for after-ripening time post physiological maturity (PM) is important when determining the relationship between PHS and malting quality. Germination/dormancy traits are not static (Rooney et al. 2021; Woods and McCallum 2000). The malting quality of lines exhibiting little to no dormancy changes with after-ripening (Frančáková et al. 2012; Woonton et al. 2005), though it also seems to plateau for most traits at some point post PM. Changes in malting quality over time would be expected to be more pronounced in lines with seed dormancy. The environmental conditions, both in field and post-harvest storage, also play a significant role in determining dormancy (Briggs et al. 1994; Gong et al. 2014; Gualano and Benech-Arnold 2009), which makes controlling for environmental effects imperative.

There are several different classes of malt each with their own target quality profiles, malt for all-malt brewing, adjunct brewing, and distilling (*Malting Barley Breeding *Guidelines 2019). Only the first two will be discussed here. Adjunct brewing utilizes other, non-enzymatically active starch sources (i.e., rice, corn) as well as malted barley in the mash (Poreda et al. 2014). The adjunct malt is characterized by high DP, AA, and FAN to compensate for the “dilution” of these components when other, non-enzymatically active starch sources are added to the mash. “Canadian-style” malt is an adjunct malt known for high values for AA, DP, and FAN, even among adjunct malts. All-malt brewing is characterized by lower values of AA, DP, and FAN. There is evidence in Canadian spring malting barley that PHS susceptibility is associated with adjunct style malts (Mohammadi et al. 2015; Sweeney et al. 2021a, b). It may be that barley intended for adjunct style malts must generally be PHS susceptible to achieve the AA, DP, and FAN specifications, while barley intended for all-malt brewing will not be penalized by PHS resistance.

Direct selection for complex traits is often confounded by their correlation to secondary traits. Achieving gains when the primary trait is negatively correlated with undesirable/antagonistic secondary traits is inherently difficult (Bernardo 2010; Sleper and Bernado 2018; Neyhart et al 2019). For example, selection for plant height in maize typically results in reduced yield (Chi et al. 1969). However, non-antagonistic secondary traits can act as a proxy for gains toward the primary trait, particularly when phenotyping the primary trait is difficult or expensive (Richards et al 2010; Wasson et al 2012). Malting quality is a complex trait and difficult and expensive to collect (Schmitt and Budde 2011). Leveraging correlated secondary traits that are less expensive would likely be valuable in barley breeding. There is evidence that germination traits like germination rate (GI) may be correlated with malting quality traits (Frančáková et al. 2012); however, this correlation has not been examined in a large number of lines, or within a breeding population. Thus the objectives of this study were to (1) examine the genetic basis of malting quality traits using genome wide association (GWA) analysis to reveal common associations between malting quality traits and germination traits, (2) examine the relationships between PHS resistance, temporal changes in germination during after-ripening, and malting quality in a germplasm base derived from Canadian, western United States, and European 2-row spring malting barley varieties, and (3) examine the effect of genomic or phenotypic selection against PHS susceptibility on the mean and genetic variance of malting quality traits.

## Materials and methods

### Spring barley germplasm

The population development for the lines used in this study were described in detail in Sweeney et al. (2020) and Sweeney, Rooney, and Sorrells (2021). Parents “Conlon” (PI 597,789), “Pinnacle” (PI 643354), “ND Genesis” (PI 677,345) (North Dakota State University), “Craft” (PI 646,158) (Montana State University), “KWS Tinka” (PI 681,721) (European variety from KWS), “Newdale” (Legge et al. 2008), and “Bentley” (Juskiw et al. 2009) (Canadian varieties) were crossed to the Canadian variety “AAC Synergy” (Legge et al. 2014). A stratified random sampling of the seven families formed the base population (C0). Two individuals from all families except the “AAC Synergy” by “Newdale” and “Bentley” families were selected with genomic selection or phenotypic selection and intermated to create the cycle 1 genotypically (C1G) selected lines and the cycle 1 phenotypically (C1P) selected lines. Selection was performed using an index selection scheme heavily weighted for PHS resistance, moderately for spot blotch, and lightly for heading date, leaf rust, grain protein, and height. In the early generations of C1G the top 15 F_2_ individuals were selected with genomic selection using an updated index with higher weights for disease resistance and intermated to create cycle 2 genotypically (C2G) selected lines. 88 lines derived from the top performing 25% of F_2_ individuals in C1G based on genomic selection using the initial index were used as the C1G. A random subset (*n* = 108) of the C1P were used as the C1P representatives. In total the population used was comprised of the eight parents, *n* = 105 C0 (~ 15 from each half-sibling family), 87 C1G, 108 C1P, and 114 C2G lines. See Supplemental Fig. 1.

### Data collection

All lines were grown in 2019 and 2020 at two locations. Half the population (represented by *n* = 66 C0, 37 C1G, 64 C1P, 58 C2G, and the eight parental lines) was grown in one location in 2021 (Sweeney et al 2021a, b). The reduction in sample size in 2021 was due to pandemic and labor restraints; however, the genetic space was sampled as uniformly as possible. All trials were planted in an augmented block design with parents replicated across blocks and all other lines present only once. Germination traits were measured on samples from all locations, while malting quality was only measured from one location per year. Spikes were harvested at two day post PM defined on a per plot basis according to the loss of green color from the peduncle. Between 30 and 50 spikes were harvested, dried for two days at 37 C, then hand threshed and stored at −20 C to prevent after-ripening from progressing (Briggs et al. 1994). Once all lines were harvested, samples were removed from the freezer and stored in ambient lab conditions. Germination assays were conducted according to Sweeney et al. (2021a, b) using 30 barley kernels with four ml of water and two Whatman #1 filter papers in a 90 mm petri plate, replicated twice per experimental plot. Germinated kernels were counted at 24, 48, and 72 h after plating. Germination percentage (GE) was calculated as the proportion of kernels that germinated out of all kernels placed within a plate. A germination rate (GI) based on an inverse mean germination time was calculated as

1 $${\text{GI }} = \, 10 \times \left( {n_{24} + n_{48} + n_{72} } \right) \times {\text{GE}}/\left( {1 \times n_{24} + 2 \times n_{48} + 3 \times n_{72} } \right)$$

where n_24_, n_48_, and n_72_ are the numbers of kernels that germinated at 24, 48, and 72 h, respectively (Frančáková et al. 2012). These germination assays were conducted at 6, 20, 34, 48, 69, 110, and 160 days post PM and are labeled TP1–TP7. Sweeney et al. (2021a, b) showed that GI at measured at TP1 (6 days post PM) was similar to spike-wetting tests and in this study, GI at TP1 will be used as the measure of PHS resistance.

All-malting was performed at the USDA-ARS Cereal Crops Research Unit (CCRU) in Madison, Wisconsin. Malting data wer collected on grain from one trial each year in 2019, 2020, and 2021 according to Schmitt and Budde (2011) on one replicate of 2.25 g for each line at each malting timepoint. The same subset of ~ 55% of the total population was malted at two timepoints in 2020 and 2021, the first timepoint was 48 days post PM, corresponding to TP4, and the second was 110 days post PM corresponding to TP6. In 2020, the rest of the population was malted only at 110 days post PM. Batch freezing at −20 C was used to bring all lines to the appropriate after-ripening time at malting. In 2019, lines were malted between 150 and 200 days post PM without rigid control over after-ripening. Seven traits were collected per sample according to scaled ASBC methods, AA (20˚ DU), DP (˚ASBC), FAN (PPM), BG (PPM), ME (% dry basis), SP (% dry basis), MP (% dry basis), and S/T (% dry basis). Internal quality checks were included within every analysis. Two other traits, CCRU_adj and CCRU_allMalt, were calculated with the standard scoring system used by CCRU to rank barley according to end use, adjunct or all-malt brewing, respectively. This scoring system was adapted for the traits collected (see Supplemental Table 1) and used to develop total quality scores per line. A higher score within a category indicated good quality for that style of malt. Based on the results of variance partitioning to detect changes in malting quality over time (described below) the 2019 trial that was malted without rigid after-ripening control was included in this study. This group was comprised of 99 C0, 74 C1G, 81 C1P, 106 C2G, and the eight parental lines.

### Genetic data

The genetic data in this study was generated and described by Rooney et al. (2021). All lines were genotyped with the 50 k SNP chip (Bayer et al. 2017) at the USDA Small Grains Genotyping Laboratory in Fargo, ND. Marker data with > 10% heterozygosity and minor allele frequency < 0.05 were filtered out. Redundant markers were eliminated using LD pruning with the SNPrelate R package (Zheng et al. 2012) using a 25,000-bp sliding window and a cutoff of 0.95. This resulted in 11,615 polymorphic markers used for the full population, and 11,443 markers when the half population was used. Two KASP markers, AlaAT_L214F and MKK3_E165Q, (Sweeney et al. 2021a, b) were included in the genetic data. These markers identify the causative SNP in *HvAlaAT1* identified by Sato et al. (2016) and the SNP identified by Vetch et al. (2020) in *HvMKK3*. Sweeney (2021) used the MKK3_E165Q KASP marker and 50 K marker JHI-Hv50k-2016–367,342 from the 50 k SNP chip to distinguish three alleles of *HvMKK3* present in this population. These alleles were called the “highly non-dormant” (MKK3_N*_), the “non-dormant” (MKK3_N_), and the “dormant” (MKK3_D_) based on their phenotypes.

### Statistical analysis

All data analyses were performed within the R statistical language version 4.0.3 (R Core Team 2020) in the RStudio IDE (RStudio Team 2021).

#### Initial variance partitioning, time of malting, and heritability

Raw malting quality data collected in duplicate (SP, ST, ME) or triplicate (FAN, BG, AA, DP) technical replicates per plot per timepoint, were used to fit models. Malting timepoint and year effects were included as fixed effects in all models. Variance components across years, within year, and within year-timepoint combination were estimated. For all traits per timepoint per year, a model of the form

2 $$Y_{{{\text{ie}}}} = \, p_{i} + \, e_{{{\text{ie}}}}$$

was fitted where *Y*_ie_ is a vector of the malting quality trait phenotypes, *p*_i_ as a random effect for plot *i* ~ *N*(0,σ_pl_^2^), and *e*_ie_ is normally distributed error. The ratio of the plot variance to the total variance (*σ*_pl_^2^ + *σ*_error_^2^) corresponds to a technical heritability or repeatability of the malting quality assays.

Second, on a per year (for 2020 and 2021) per trait basis, the relative importance of the per line by timepoint deviation from a main line effect was assessed. Models of the form

3 $$Y_{{{\text{ijk}}}} = \, T_{i} + \, g_{j} + \, g:T_{{{\text{ij}}}} + \, e_{{{\text{ijk}}}}$$

were fit to each trait where *Y*_ijk_ is a vector of the malting quality trait phenotypes, *T* is a fixed effect for timepoint *i*, *g* is a random effect for line *j* ~ *N*(0,*σ*_*g*_^2^), *g*:*T*_ij_ is a random effect for timepoint *i*’s interaction with line *j* ~ *N*(0,*σ*_*g*:TP_^2^), and e_ijk_ is normally distributed error. Most lines were only malted once per timepoint, therefore residual errors were approximately equal to those from the first model. The *σ*_*g*:TP_^2^ contained the variance explainable both by the malting timepoint specific changes that occurred to each line, but also a plot level error variance (*σ*_pltE_^2^), i.e., the variance of each trait, separate from the malting assay’s extraction error variance (variance of repeated measurement of the same sample) as estimated within the error term in model 2, when the same plot is sampled repeatedly and malted. If the assumption is made that the plot level variance is identical at each timepoint for each line and these variables are independent, then

4 $$\sigma_{{g:{\text{TP}}}}^{2} \approx \sigma_{{\Delta g:{\text{TP}}}}^{2} + \, \sigma_{{{\text{pltE}}}}^{2}$$

where *σ*_Δ*g*:TP_^2^ is the variance associated with phenotypic change across timepoints. *σ*_pltE_^2^ was estimated with an internal check, c.v. “Tradition,” (PI 612,442 Busch Agriculture Resources, 2003) that was included and replicated every 15 samples with grain sourced from the same lot. The variance of Tradition on a replicate plot basis was estimated as the variance of Tradition trait values minus the error variance associated with the assay (from model 2 or 3). *σ*_Δ*g*:TP_^2^ was estimated and gives an indication of how much of the *σ*_*g*:TP_^2^ was due to plot level error versus changes in the malting quality that occurred during after-ripening. This is an approximation, and the output should be treated with caution.

Third, per trait (including all years, 2019, 2020, and 2021) models of the form

5 $$Y_{{{\text{yijk}}}} = \, E_{y} + \, T_{i} + \, E:T_{{{\text{yi}}}} + \, g_{j} + \, g:T_{{{\text{ij}}}} + \, e_{{{\text{yijk}}}}$$

were fitted where *Y*_yijk_ is a vector of the malting quality trait phenotypes, *E* is a fixed effect for year *y*, *T* is a fixed effect for timepoint *i*, *E*:*T* is a fixed effect interaction term for year-timepoint combination *y*_*i*_, *g* is a random effect for line *j* ~ *N*(0, *σ*_*g*_^2^), g:*T* is a random effect for each line *j*’s interaction with timepoint *i* ~ N(0, *σ*_*g*:TP_^2^), and e is normally distributed error ~ *N*(0, *σ*_error_^2^). 2019 data were assigned a value of TP6 for the malting timepoint based on the results from the previous models. Heritability of traits across years was assessed as

6 $$\sigma_{g}^{2} /\left( {\sigma_{g}^{2} + \, \sigma_{{g:{\text{TP}}}}^{2} + \, \sigma_{{{\text{error}}}}^{2} /y} \right)$$

where y is the number of years (*y* = 3). All the previous mixed model analyses were performed using the lme4 R package (Bates et al. 2015).

#### Estimation of line effects and genome wide association analysis

The raw malting quality data as before were used as the input. For 2020 and 2021 on a per year basis, model 3 was reparametrized as a fixed effect model. Line best linear unbiased estimators (BLUEs) at each timepoint (labeled as “TP4” or “TP6”) were then extracted from the model and used as the input to GWA per timepoint. A second model was fit per year per trait of the form

7 $$Y_{{{\text{ijk}}}} = \, T_{i} + \, G_{j} + \, e_{{{\text{ijk}}}}$$

where *Y*_ijk_ is a vector of the malting quality trait phenotypes, *T* was a fixed effect for timepoint *i*, *G* was a fixed effect for the average line effect *j* across timepoints, in effect treating the two timepoints as replicates, and e was normally distributed error. Line BLUEs were extracted from the model (labeled as “TP4/TP6” for 2020 and 2021) and used as the input to GWA per year.

For 2020 and 2021 (labeled as “20/21”) and for 2019, 2020, and 2021 (only TP6, labeled as 19/20/21) model 5 was reparametrized as a fixed effect model with a heterogeneous error variance per year and line BLUEs extracted for each timepoint (labeled as “TP4” or “TP6”). A second model was fit per trait of the form

8 $$Y_{{{\text{yijk}}}} = \, E_{y} + \, T_{i} + \, E:T_{{{\text{yi}}}} + \, G_{j} + \, e_{{{\text{yijk}}}}$$

where *Y*_yijk_ is a vector of the malting quality trait phenotypes, *E* was a fixed effect for year *y*, *T* was a fixed effect for timepoint *i*, *E*:*T* was a fixed effect for year *y*’s interaction with timepoint *i*, *G* was a fixed effect for the line effect *j*, and e was a normally distributed heterogeneous error variance per year. BLUEs per line were then extracted (labeled as “TP4/TP6”) and used as the input to GWA. Lastly, model 8 was fit to all the data (2019, 2020, and 2021 together) to estimate line effects overall years, labeled as ‘Combined’. See Supplemental Table 2 for a table summary of GWA analyses performed. Any models with heterogeneous variance components were fit with ASReml-R (Butler et al. 2018).

GWA analysis was performed within the GAPIT framework (Lipka et al. 2012) with multiple locus mixed model (MLMM) (Segura et al. 2012). The MLMM model includes highly significant markers as fixed effects within the model in a forward backward stepwise regression, which can allow for the identification of multiple factors in close physical proximity to be distinguished. Two principal components from eigenvalue decomposition of the relationship matrix were used as fixed effects in the model to account for population structure and kinship was used as a random effect to account for relatedness. A significance threshold of p-value < 5E-5 was used.

#### Genetic correlations

Genetic correlations between germination traits and malting quality traits were calculated using bivariate mixed models. Plot level means were used as the input to these models to allow for paired observations. For germination traits technical replicates were averaged across plots per timepoint and for malt traits technical replicates were averaged across or within malting timepoints per plot. All models were of the form

9 $$Y_{{{\text{tyj}}}} = \, E_{{{\text{ty}}}} + \, g_{{{\text{tj}}}} + \, e_{{{\text{tyj}}}}$$

where *Y*_tyi_ is a two column by *n* row matrix of observed phenotypes of trait *t* with each phenotype corresponding to a column,* E* is a fixed effect for each trait *t* in each year *y*, *g* was a random effect for line *j*’s effect for trait *t*, and e was heterogeneous, normally distributed error for each trait *t,* in each year *y.* g was assigned a general heterogeneous correlation structure, with the variances of each trait on the diagonal of the matrix and the covariances between each trait on the off-diagonals. Model 9 was compared to an equivalent model with a diagonal variance structure assuming no correlations between traits for the line effect with a likelihood ratio test to assess improvements in model fit and significance of correlation. *HvMKK3* is associated with differences in germination phenotypes and large effect loci can lead to changes in marker effects genome wide (Rooney et al. 2022). It is also possible substantial correlations can be driven by a large effect locus. All correlations were also calculated on a per *HvMKK3* allele basis. All genetic correlations were estimated with ASReml-R. Eigenvalue decomposition of the phenotypic correlation matrix calculated from the scaled and centered BLUEs of the malting quality traits, and germination traits across *HvMKK3* alleles and per allele was used to generate biplots to show trait relationships (Pauli et al. 2018).

#### Correlated response to selection

Since all-malted lines were part of a selection experiment (Sweeney et al. 2021a, b) the correlated response to selection for the malting quality traits was examined. PHS resistance and grain protein content were likely the only two factors included in the selection index that may have influenced malting quality. Correlated response to selection was examined across years, treating malting quality timepoints as replicates by combining years 2019, 2020, and 2021. Models of the form

10 $$Y_{{{\text{ycijk}}}} = \, E_{y} + \, C_{c} + \, T_{i} + \, E:T_{{{\text{yi}}}} + \, T:C_{{{\text{ic}}}} + \, E:C_{{{\text{yc}}}} + \, g_{j} + \, e_{{{\text{ycijk}}}}$$

where *Y* is a vector of malting quality phenotypes, *E* is a fixed effect for year *y*, *C* is a fixed effect for selection cycle *c*, *T* is a fixed effect for timepoint *i*, *E*:*T*, *T*:*C*, and *E*:*C* are all fixed interaction terms between cycle, timepoint, and year, and g is a random effect for line *j* with a heterogeneous error structure ~ N(0, *σ*_*g*_^**2**^) where *σ*_*g*_^**2**^ was a diagonal matrix with a unique genetic variance per cycle of selection. Error was distributed as ~ N(0, *σ*_**error**_^**2**^) where *σ*_**error**_^**2**^ was a diagonal matrix with a unique error variance per year. Model 10 was compared to an identical model except for using a homogeneous variance structure for the random line effect, with a likelihood ratio test to assess increased model fit and changes in genetic variance between the cycles of selection. The cycle fixed effects were tested for significant differences from zero using a Wald test in ASReml-R. Kenward − Roger degrees of freedom were estimated from the *wald()* object and used within a t-distribution to determine a p-value. The test statistics (t values) were estimated as the cycle fixed effect estimate divided by its standard error.

## Results

### Variance partitioning and heritability

Malting quality trait distributions per year and timepoint are shown in Supplemental Fig. 2 and Supplemental Table 3. Technical heritability for all traits was high, rarely decreasing below 0.80 within any timepoint, year combination (Supplemental Table 3). Analysis of model 3, variance of the Tradition malt check, and the heritability on a per year basis are shown in Supplemental Table 4. Heritability on a per year basis was generally moderate to low with BG being the lowest in both years at about 0.32, and SP being the highest in both years at 0.6 to 0.7. Estimation of *σ*_Δ*g*:TP_^2^ for each trait within year was mostly successful, but the assumptions led to a negative *σ*_Δ*g*:TP_^2^ for BG in 2020 and FAN in 2021. Relative importance of *σ*_Δ*g*:TP_^2^ was assessed in relation to the line variance of model 3 as *σ*_Δ*g*:TP_^2^/ σ_g_^2^ (ratio 2, Supplemental Table 4) with the resulting values ranging from 1.15 for 2021 BG to 0.082 for 2021 SP. Most of the ratios were less than 0.5. Heritability across years (model 5) was 0.52 for AA, 0.37 for BG, 0.31 for CCRU_Adj, 0.17 for CCRU_allMalt, 0.59 for DP, 0.59 for FAN, 0.49 for ME, 0.68 for MP, 0.73 for SP, and 0.39 for S/T. Supplemental Table 5 contains variance components from model 5.

### Combined GWA analyses results

**Fig. 1:**
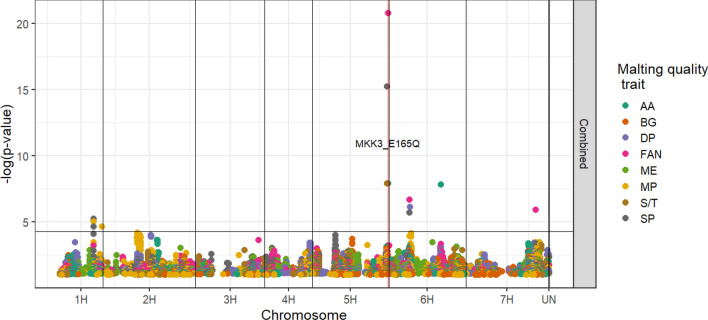
Manhattan plot for marker trait associations from the combined analysis (2019, 2020, 2021, treating malting timepoints as replicates). Chromosome is shown along the* x*-axis, −log10* p*-value is shown along the* y*. Color corresponds to the malting trait. The position of MKK3_E165Q is shown with a red line (colour figure online)

Figure 1 displays a Manhattan plot of marker trait associations (MTAs) for the combined dataset (2019, 2020, and 2021 together, treating timepoint as replicates). Supplemental Table 6 displays all MTAs. For the combined dataset treating malting quality timepoints as replicates, AA and FAN were most strongly associated with MKK3_E165Q. Soluble protein and S/T were most strongly associated with a marker in the *SD2* region approximately 2.4 Mbp proximal to the *HvMKK3*. This SNP was in moderate LD (*r* = −0.76) with MKK3_E165Q and was less correlated with the other alleles of *HvMKK3* (MKK3_D_: −0.07, MKK3_N_: −0.56). Traits DP, FAN, MP, and SP were associated with a region on chromosome 6H. All significant markers in the region were in high LD (r > 0.94) centering around the 50 k marker JHI-Hv50k-2016–385,463. Alpha Amylase was associated with a different region on chromosome 6H. Free amino nitrogen was associated with one marker near the distal end of chromosome 7H, and SP and MP were associated with a region near the distal end of chromosome 1H.

### Other significant marker trait associations

The other combinations of year/malting quality timepoint generally repeated these results, except for a few markers reaching significance in only one year or year group (Supplemental Table 6). In 2019 and 2021, AA MTA were found on the chromosome 7H and end of chromosome 2H, respectively. In 2020, 2021 and 2020/2021 only for TP4, a region on chromosome 5H at 517 Mbp was significantly associated with BG. A DP MTA was found with a marker in almost perfect LD (correlation = 0.998) to the chromosome 6H AA MTA in the combined dataset in 2020 and the 2020/2021. Diastatic power also had MTA with markers on chromosomes 4H and 2H in 2021. The marker on chromosome 2H was not in LD (*r* = 0.01) with the MTA found for AA on chromosome 2H in 2019. For FAN, MTA were found on the proximal end of chromosome 4H and near the middle of chromosome 3H in 2020/2021 and 2020, respectively. A ME MTA was found on the middle of chromosome 2H for 2020 and 2020/2021. For MP, a MTA centering at 365 Mbp on chromosome 2H was found for TP6 in the combined 19/20/21 dataset. For SP, a marker that colocalized with the FAN MTA (*r* = 0.97) in the combined analysis on chromosome 7H was significant in 2021. Another FAN MTA was found near the middle of chromosome 5H for 2020/2021. This marker was unrelated (*r* = −0.17) to the BG MTA found in the region. A S/T MTA at 527 Mbp on chromosome 3H for TP6 in the combined 19/20/21 dataset.

### Genetic correlation

**Fig. 2:**
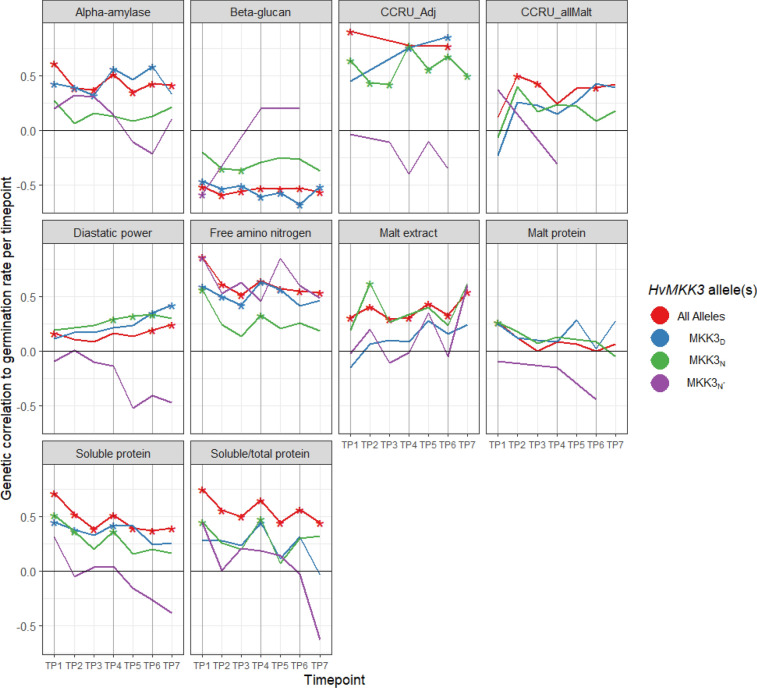
Change in genetic correlations of germination rate (GI) and malting quality trait over time.* X*-axis is timepoint,* Y*-axis is the correlation, plotted is the genetic correlation of the malting quality and germination rate at that timepoint across and within *HvMKK3* alleles. Traits are listed in the facet header. Timepoints TP1, TP4, and TP6 (shown with darker vertical lines) are calculated with three years of data while the rest are calculated with two years. Points plotted with an asterisk indicate significant increases in model likelihood (*p < 0.01) and significance of correlation

Genetic correlations of malting traits with GE or GI per timepoint were similar until later timepoints when the genetic variance of GE declined as most lines reached 100% germination (Supplemental Table 7). Only TP1, TP4, and TP6 had germination data for three full years and will be discussed here. Figure 2 shows results from the other timepoints with two years of data as well. When considering all alleles of *HvMKK3* simultaneously, high correlations were observed between the malting traits and GI TP1 and declined or remained of similar magnitude for the subsequent timepoints. Diastatic power was weakly (*r* < 0.2) correlated with GI TP1 and TP6, but not GI TP4. Free amino nitrogen, S/T, SP, and AA were correlated with GI TP1 (*r* = 0.85, 0.75, 0.72, and 0.62, respectively) and retained high correlations to GI TP4 and TP6. Beta glucan was moderately negatively correlated with GI TP1 (*r* = −0.517) and remained negatively correlated over time. Malt extract was weakly positively correlated with GI TP1 (*r* = 0.30). The CCRU_Adj score was strongly correlated with GI TP1 (*r* = 0.90) and retained a high correlation to the GI over time. The CCRU_allMalt was weakly correlated with GI TP6 (*r* = 0.20 to 0.40). Malt protein was weakly correlated with GI TP1 (*r* = 0.26). Supplemental Table 7 shows results for variations of model 9 including different groups of years or malting timepoints. These results were similar to the results in Fig. 2.

Within each *HvMKK3* allelic groups multiple significant correlations were detected, particularly with FAN, SP, S/T, and AA. For MKK3_N*_, FAN was correlated with GI TP1 (*r* = 0.85) and BG was negatively (*r* = −0.58) correlated with GI TP1. For MKK3_N_, SP, S/T, and FAN were correlated (0.3 < *r* < 0.5) with GI TP1 and TP4. Germination rate at TP1 was not significantly correlated with AA (*r* = 0.26). CCRU_Adj was moderately (*r* ~ 0.7) correlated with GI at all timepoints. BG was not significantly correlated with GI TP4 and TP6, while DP was weakly positively related to GI TP4 and TP6. For MKK3_D_ the highest number and magnitude of significant correlations were observed. GI TP1 was weakly to moderately correlated with AA, BG, FAN, and SP. These correlations generally increased in magnitude with after-ripening and remained significant. The CCRU_Adj score was strongly (*r* > 0.7) correlated with GI at TP4 and TP6. The correlation of GI TP1 with DP was not significant, but increased in strength and became weakly correlated with GI TP6.

**Fig. 3:**
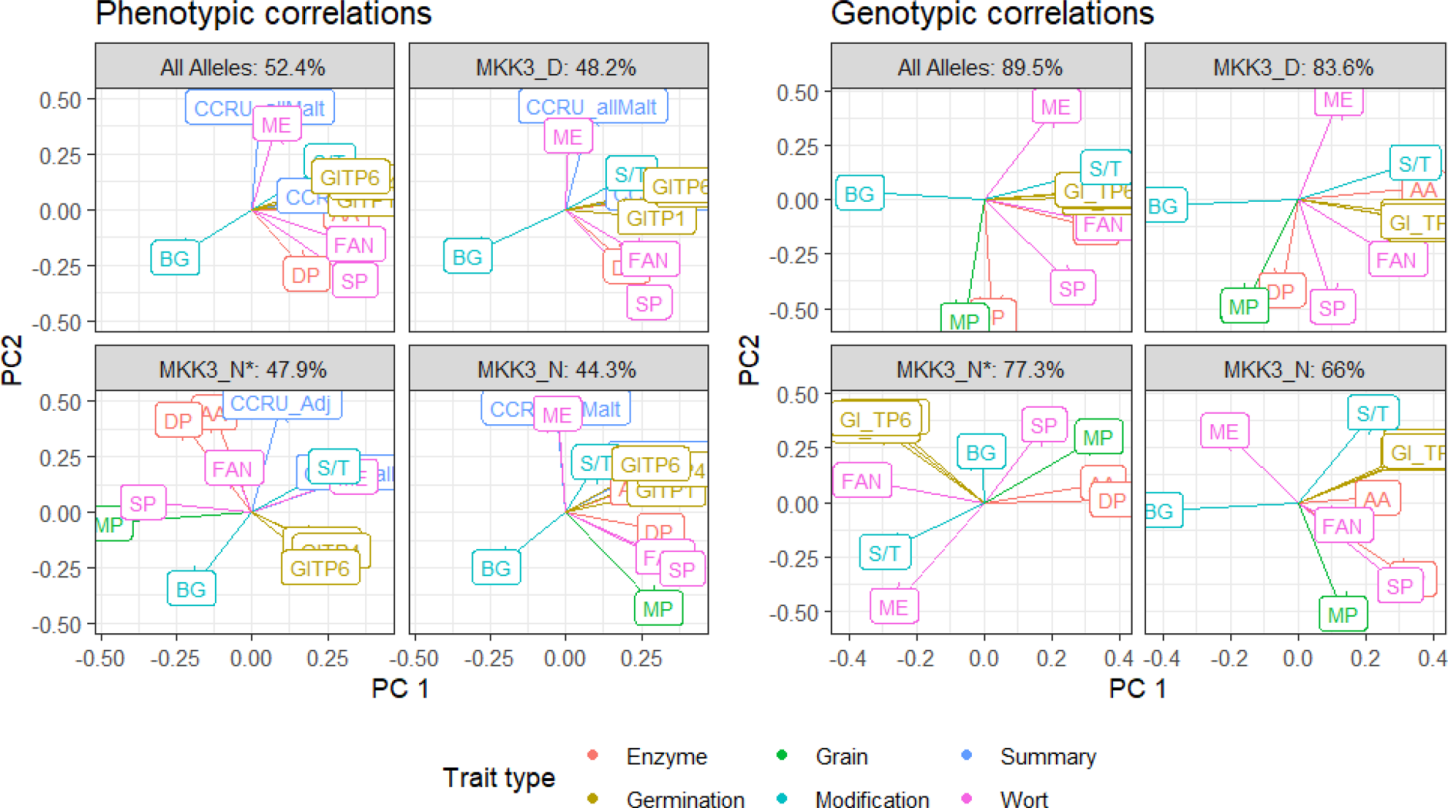
Biplots of the correlation of malting quality and germination traits. The first two principal components are the* x* and* y* axis, respectively, length of arrows indicate magnitude, and relationship of arrows to each other correspond to trait correlation. Arrows going in the same direction indicate positive correlation and opposite direction indicates negative correlation within facet. *HvMKK3* allele is labeled along the top facet, while the amount of variance explained by the first two principal components is shown beside each allele label. Biplots of the phenotypic correlations are on the left, while the genetic correlations are shown on the bottom. Colors corresponds to trait type (colour figure online)

Phenotypic and genetic correlations between the malting quality traits across all *HvMKK3* alleles and within allele are shown in Supplemental Table 8. Overall, there were weak to moderate correlations between most malting traits, with especially strong correlations between FAN and SP. Biplots (Fig. 3) give an indication of the relationships across and within *HvMKK3* alleles. In general DP and AA were related, as well as the germination traits and FAN, SP, and S/T. BG was generally negatively or unrelated to all other traits within the first and second principal components. The strength and type of the relationships changed across the *HvMKK3* alleles.

### Response to selection

**Fig. 4:**
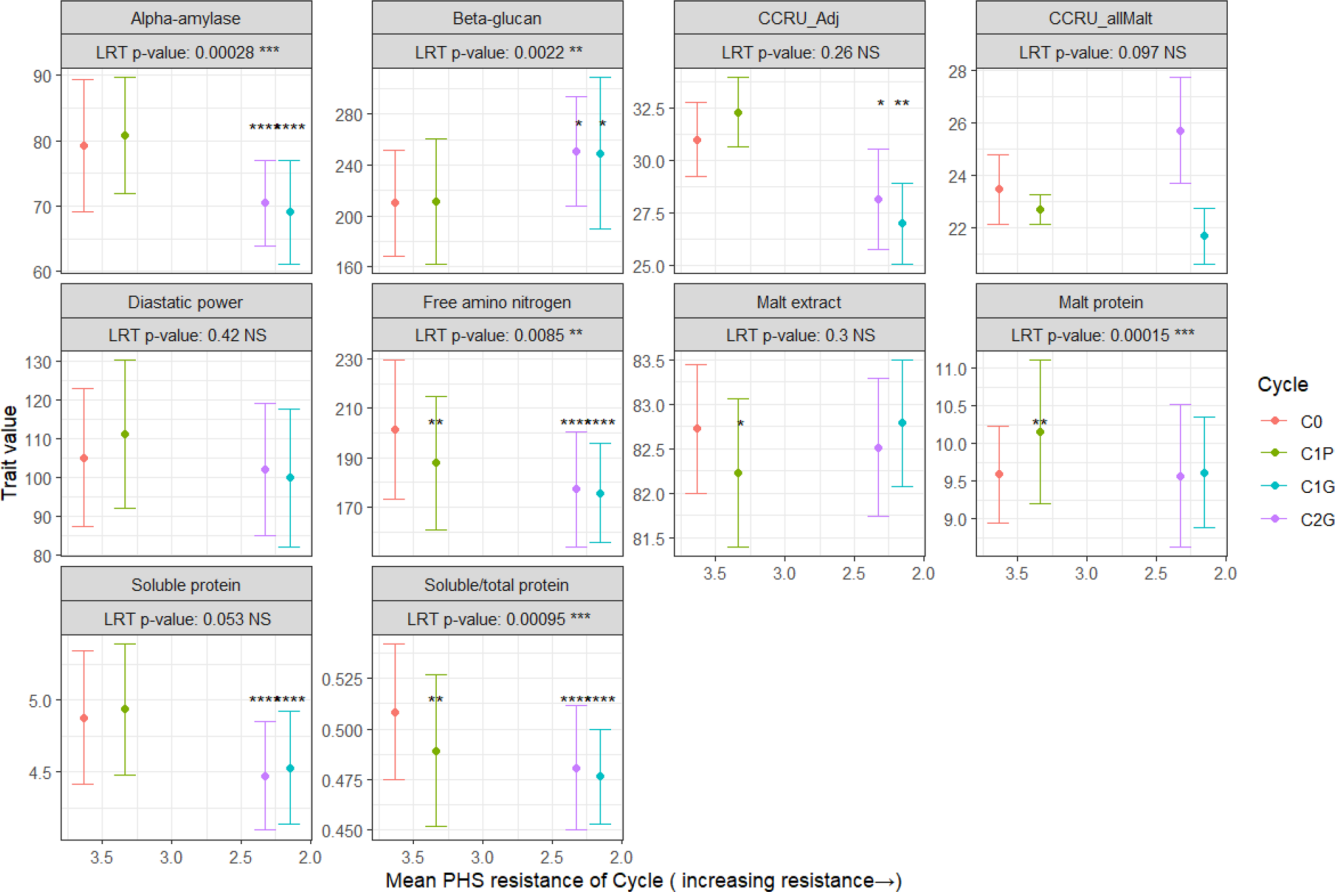
Correlated response to selection within each cycle. Traits are listed as the title of each facet. The* x*-axis is the average preharvest sprouting (PHS) resistance each cycle, a lower number indicates better PHS resistance, and the* y*-axis shows the malting quality trait value. The mean and the standard deviation are displayed as the point (mean) ± two standard deviations (the square root of the cycle genetic variance by model 10). Likelihood ratio test (LRT) results for each trait are shown below the trait label on each facet. The population and selection scheme used is shown in the diagram. Black asterisks indicate significant cycle fixed effects. (****p < 0.0001, ***p < 0.001,**p < 0.01,*p < 0.05)

Figure 4 shows the correlated response to selection on the malting quality traits in the C0 (base) population. Alpha amylase showed significantly lower values in C1G and C2G, as well as significant changes in variance. Beta glucan showed significant increases from the base population for C1G and C2G and significant changes in variance. CCRU_Adj showed a significant decrease in score within C1G and C2G. Diastatic power and the CCRU_allMalt score showed no significant changes for cycle means or variances. Free amino nitrogen showed a significant decrease for all cycles, with C1G and C2G changes being greatest, as well as changes in the variance across cycles. Malt extract showed only a slight decrease in C1P compared to the base with no changes in variance. Malt protein showed only a small and significant increase within the C1P lines and significantly different variances. Soluble protein showed a significant decrease in C1G and C2G while retaining similar genetic variances per cycle. Soluble over total protein showed a significant decrease in C1P, C1G, and C2G with differences in genetic variances between cycles.

## Discussion

The three primary objectives of this study were to examine 1) the genetic basis of malting quality traits using GWA analysis to find common associations with dormancy loci, 2) the genetic correlations between PHS resistance, germination traits over time, and malting quality, and 3) the effect of genomic or phenotypic selection against PHS susceptibility on malting quality traits across two cycles of selection. There were common MTAs for PHS susceptibility (Sweeney et al. 2021a, b), AA, and FAN with the most significant marker being the putative mutation in *HvMKK3* responsible for loss of seed dormancy (Vetch et al. 2020). Traits AA, CCRU_Adj, FAN, ME, SP, and S/T had significant positive genetic correlations to the PHS proxy trait GI TP1 within and across *HvMKK3* allelic groups. Lastly, correlated responses to selection for PHS resistance were significant, especially for the traits that had significant genetic correlations to PHS resistance. Selection against PHS led to a reduction in the CCRU_Adj score.

### Initial variance partitioning and timepoint treatments for malting

For most of the traits and lines the error variance of the assays used was too large to detect any small changes in malting quality that may have occurred between the malting timepoints (Supplemental Tables 3 and 4) on an individual line basis. The magnitude of the plot level error (*σ*_pltE_^2^) variance estimated indicated a relatively low importance for most traits of the *σ*_Δ*g*:TP_^2^, as the σ_g_^2^ generally was at least double *σ*_Δ*g*:TP_^2^ (supplemental Table 4, ratio 2), and that the TP:line deviations were mostly caused by plot level assay error (supplemental Table 4, ratio 1), rather than large changes in malting quality between 48 and 110 days post PM. This was likely due to the small quantity of grain sampled. Rooney et al. (2021) also reported that most of these lines reached a close-to asymptotic germination value before 48 days post PM with smaller changes occurring between 110 and 160 days post PM. For this reason, the 2019 malting quality data collected in a less controlled manner (malted between 150 and 200 days post PM) was added to this study and assigned the malting quality timing value of TP6. Changes in malting quality that occurred between 110 and 200 days post PM were likely too small to detect using these methods. This is also similar to (Woonton et al. 2005) who found that values for most traits plateaued after a certain period of after-ripening. Wort BG content was one of the exceptions in that study and may be here as well, as high BG values in 2021 at the first malting timepoint often significantly decreased by the second malting timepoint (Supplemental Fig. 2, Supplemental Table 3). Since on an individual line basis changes could not be detected, but on aggregate (examining all lines) there were significant differences between timepoints, malting timepoint was effectively treated as a replicate with a fixed effect. Results on a per timepoint and per year basis for GWA are presented in the supplemental material but any novel MTAs coming from a single timepoint/year combination should be treated with caution. The assay developed by Schmitt and Budde (2011) called for two replicates of 2.25 g of barley to be malted and analyzed, here for most lines on a per timepoint/plot basis only one replicate was malted. This was due to the high correlations we found between samples malted twice which led to a prioritization of replication of alleles (hence more individuals) with fewer replications per individual. Most lines were malted twice, just at two different timepoints.

### MKK3_N*_ likely has pleiotropic effects on several malting quality traits

In the combined GWA analysis (Fig. 1, supplemental Table 6) the *SD2* region was shown to be significantly associated with AA, FAN, SP, and S/T. In particular, the MKK3_N*_was associated with higher AA and FAN. This region on chromosome 5H has been implicated numerous times for similar malting quality traits in other studies (Beattie et al. 2010; Belcher et al. 2018; Castro et al. 2010; Li et al. 2003; Lin et al. 2009; Marquez-Cedillo et al. 2000; Matthies et al. 2014; Mohammadi et al. 2015; Pauli et al. 2018; Sweene et al. 2021; Zhang et al. 2011; G. Zhou et al. 2016; T. Zhou et al. 2011). The only recent study that has not detected a significant association in the region performed mapping within United Kingdom varieties (Looseley et al. 2020) which may not be segregating within the *SD2* region or the alleles that are present do not have different effects on malting quality. The common association of AA and FAN to the putative causative mutation in *HvMKK3* (genotyped by the MKK3_E165Q KASP marker) is strong evidence that *HvMKK3*, especially the MKK3_N*_ allele, has pleiotropic effects on malting quality.

Surprisingly, for the combined GWA, SP and S/T were not most strongly associated with the MKK3_E165Q mutation or another SNP in tighter linkage with it. They most strongly associated with a SNP 2.4 Mbp away from the mutation only moderately correlated with the MKK3_N*_ allele. The MLMM model adds markers stepwise as fixed effects to the linear model used in significance testing. Simpler models such as the mixed linear model from Yu et al. (2006) show the *SD2* region broadly as highly significant, including the MKK3_E165Q mutation. This may indicate increased measurement error for SP or S/T. Soluble over total protein had a low across year heritability, but SP had the highest across years heritability (Supplemental Tables 3–5). For other combinations of the year-timepoint data SP was significantly associated with the MKK3_E165Q marker (Supplemental Table 6) which could suggest error within particular years or timepoints. Another plausible explanation would be the presence of two genes in the larger *SD2* region affecting malting quality, *HvMKK3* which has effects on AA and FAN, while another unknown gene affects SP and S/T. Additional experiments to knock out the *HvMKK3* alleles would add stronger evidence for pleiotropic effects on malting quality traits.

The nature of these pleiotropic effects remains unclear. The MKK3_N*_ allele leads to an extremely high germination rate. Germination rate affects timing and amount of enzymes production, starch breakdown, and modification. MKK3_N*_ could lead to higher modification (higher S/T, lower BG, higher enzyme activities, and FAN) because of the allele per se or because it mediates higher germination rate. If germination rate leads to the changes instead of the allele per se, modifying the malting schedule or the germination rate with exogenously produced plant hormones could allow maltsters to fine tune the malt profile without the risk of a PHS susceptible allele of *HvMKK3*.

### Other significant markers DP, FAN, SP, and MP

The common association of BG, DP, FAN, MP, and SP all with a single region on chromosome 6H is similar to results in in Montana State University germplasm (Pauli et al. 2018) and six-row germplasm (Belcher et al. 2018). A gene cluster or pleiotropy of a single gene in the region could lead to these associations. Further fine mapping would be required to resolve this question. The locus does not co-locate with any germination related loci found in Rooney et al. (2021) or Sweeney, Rooney, and Walling et al. (2021). The AA association on Chr 6H at 524 Mbp is in a similar region to the AA association identified by Looseley et al. (2020) and the SP association on Chr 1H may be related to that identified by (Matthies et al. 2014); however, both of these studies focus on European malting barley. The FAN association on Ch7H may have been identified previously in a Canadian by Australian barley cross (G. Zhou et al. 2016) and most significant regions are also identified within and across North American breeding programs by Mohammadi et al (2015), which is in conformity with the genetic background of the population.

### Genetic correlations and implications for selection

Since single large effect loci can rapidly reach fixation within a breeding program and drive genetic correlations if they act pleiotropically, the genetic correlations across and within *HvMKK3* allelic groups were examined. Within allele there were significant, moderate to high correlations of AA, FAN, SP, and S/T to GI TP1. This correlation was often lower within allele group than across all alleles, but still positive and often significant. A component of the genetic correlation was associated with *HvMKK3*, but linkage or pleiotropy of smaller effect quantitative trait loci across the genome still leads to significant genetic correlations within allelic group. Consequently, if malting occurs during this period, selection for increased values of AA, FAN, and/or S/T, will result in increased PHS susceptibility. The result of this is apparent within Canadian and western US spring barley breeding programs. These production regions have historically supported adjunct brewing and varieties for this region are known to be PHS susceptible (Sweeney et al. 2021a, b; Vetch et al. 2020; Zhang et al. 2011) and enriched for MKK3_N*_. Recurrent selection for high FAN and AA in these programs enriched their varieties for the PHS susceptible MKK_N*_ *HvMKK3* allele and further increased PHS susceptibility in that allelic group by continuing selection for increased FAN.

Lines carrying the MKK3_N*_ allele exhibited the fewest significant correlations with germination traits. The malting and germination assays within this allele may not have been sensitive enough to detect small variation in traits, or these traits are not significantly related to germination. The phenotypic and genetic relationship between germination traits and malting quality appear to be different across alleles. Within the MKK3_N_ and MKK3_D_ allelic groups and across all alleles, GI correlations to DP increased in strength over time. For DP germination at a specific malting time is more informative than its PHS resistance in the field. This may be because of two factors. First, a lower GI may indicate that some kernels have failed to germinate due to disease. Germination is affected by oxygen concentration and abscisic acid sensitivity in the embryo (Benech-Arnold et al. 1999, 2006). Microbial infection on the seed coat can prevent oxygen diffusion (Gaber and Roberts 1969) into the embryo and lead to increased numbers of kernels that do not germinate or germinate slowly due to water sensitivity (Crabb and Kirsop 1969) which could lead to reduced DP in the malt. Conversely, a higher GI likely leads to higher enzyme activities in the malt as the enzymes are produced more rapidly. Within the MKK3_N*_ group lines may be germinating so rapidly they are transitioning past the point of increasing amounts of enzymes and starting to degrade them. Another explanation would be that the MKK3_N*_ reduces grain storage longevity.

Within each allele ME was not significantly correlated with the germination traits; however, it was significantly related in the analysis over all alleles and could be due to the higher SP that is associated with the MKK3_N*_ allele. Malt extract is related to all soluble components so more protein in the wort from the MKK3_N*_ lines will lead to a higher ME. MKK3_N*_ was associated with a numerical increase of about 0.5 percentage SP in the wort, which could drive the weak correlation between ME and GI that is absent when examining within allele.

The significant correlations to GI at various timepoints within each *HvMKK3* allele indicate that indirect selection on GI could lead to gain for some malting traits. This was especially true within MKK3_D_ and MKK3_N_ where GI at TP4 and TP6 were moderately to well correlated (abs(*r*) > 0.4) with AA, BG, FAN, SP, or S/T at the time of malting. Previous work has shown that these germination traits are highly heritable and predictable with genomic prediction (Rooney et al. 2021; Sweeney et al. 2021a, b) and therefore may be used to simultaneously select for lines that have PHS resistance and malting quality. Multi-trait prediction and selection schemes could be useful for these traits (Bhatta et al. 2020).

### Response to selection

Given the presence of significant genetic correlations between the germination and malting quality traits selection for PHS resistance should lead to a correlated response. Selection for PHS resistance was successful (Sweeney et al. 2021a, b). Selection for decreased grain protein was imposed but did not lead to genetic gain. Malt protein is related to grain protein and the only significant difference observed was for the C1P lines, which had a slightly higher protein content on average than the base. Unless agronomic traits included in the index were genetically correlated with malting quality, selection for PHS resistance led to an unintentional response in malting quality. At the largest effect level, selection led to a reduction in the incidence of the MKK3_N*_ within the successive cycles, especially within the C1G and C2G which in turn lead to decreased cycle means for AA, FAN, SP, and S/T. If selection against PHS was continued in subsequent cycles it is likely the changes between each cycle would be reduced in magnitude. Selection against PHS susceptibility also led to lines with average CCRU_Adj scores supporting an important association between PHS susceptibility and adjunct malting quality.

### Practical application and relationship to breeding goals

**Fig. 5:**
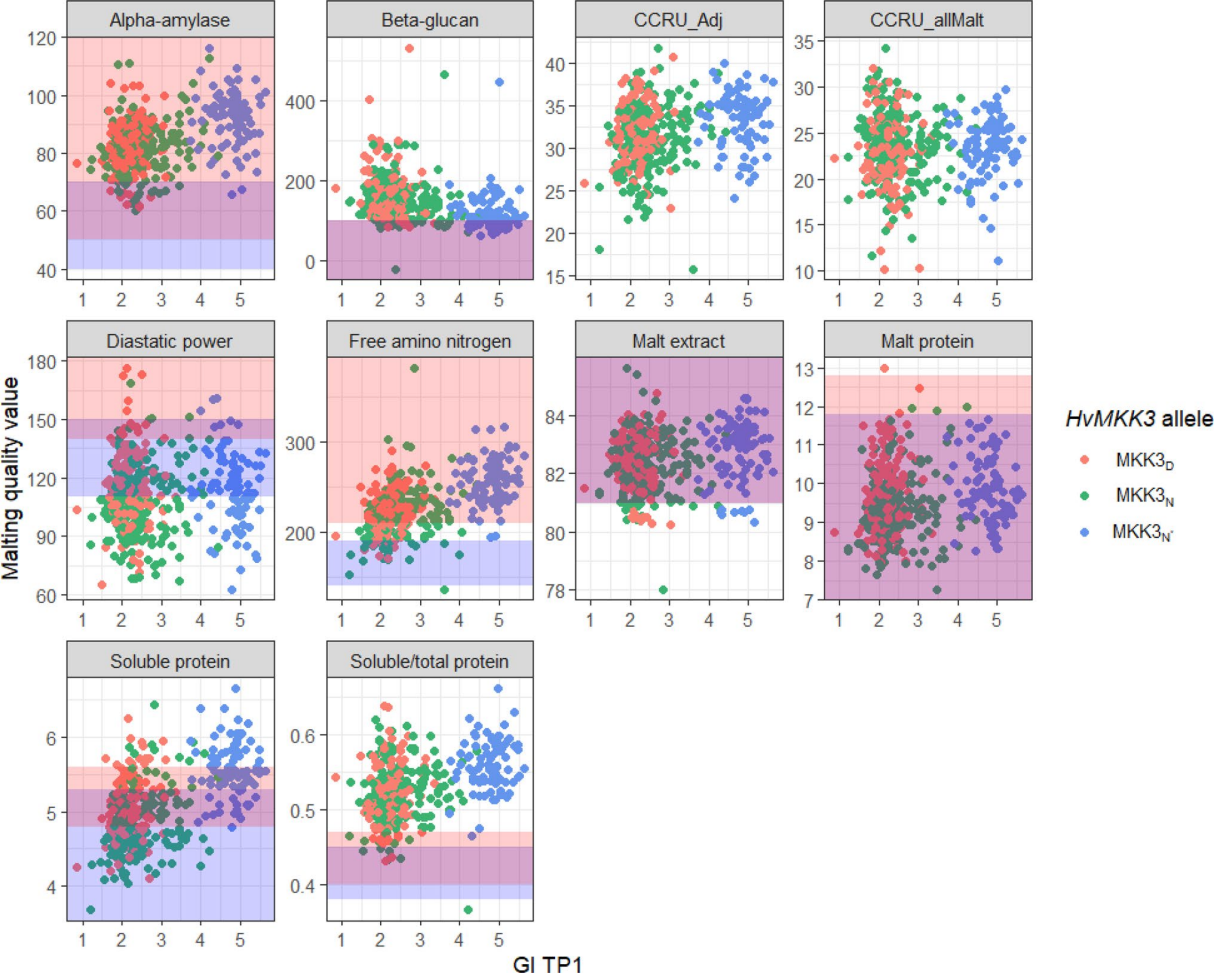
Malting quality trait values in relationship to the allele of *HvMKK3* and the germination rate at TP1 (6 days post PM) as a measure of PHS susceptibility. Malting quality traits are listed on the top of each facet, *HvMKK3* allele is shown by color, and GI TP1 is shown on the x-axis. Blue shaded graph area is the acceptable breeding target published by AMBA for all-malt brewing, red shaded area is the acceptable breeding target for adjunct brewing. Purple shading depicts overlap between the all-malt or adjunct breeding goals

For variety development, if the *HvMKK3* does not have pleiotropic effects on malting traits, malting quantitative trait loci are likely linked so tightly to *HvMKK3* at *SD2* that breaking the linkage would take a prohibitively large population size and effort. From the same perspective, within lines containing alleles PHS resistant alleles of *HvMKK3*, MKK3_D_ and MKK3_N_, there are significant correlations to germination traits, both at TP1 (PHS resistance measure) and at time of malting (47 to 200 days post PM) which could play an important role in selection decisions. The importance of correlations may be lessened because malting quality trait goals are related to absolute ranges rather than purely increased or decreased values. It is helpful to look at these values in relation to the American Malting Barley Association breeding targets (*Malting Barley Breeding *Guidelines 2019) (Fig. 5). MKK3_N*_ appears to be more associated with a trait profile for adjunct brewing while the other alleles more generally fall into the all-malt categories, especially for those traits that are highly correlated with the germination. This is quantitatively supported with the high genetic correlation between the scoring for the CCRU_adj score and the GI, while the CCRU_allMalt score is poorly or even unrelated to the GI. However, in both of these categories there are lines that meet the guidelines with any allele, albeit on the lower side of the range for adjunct malt with the non MKK3_N*_ lines, reproducing previous results (Edney et al. 2013). The exception to this is BG, which was high for all alleles. This is likely due to high values of BG in the 2019 year. Sweeney et al. (2021a, b) reported that the MKK3_N*_ allele was enriched in Canadian and western US breeding programs targeted for adjunct malting quality. MKK3_N*_ generally produces a line with a higher chance of meeting the adjunct style malting quality guidelines, strongly implicating pleiotropy of *HvMKK3* leading to the Canadian style adjunct malt.

This work was undertaken to address the demand for PHS resistant, high quality malting barleys by elucidating the relationship between PHS resistance and malting quality. There are strong genetic relationships between some malting quality traits and PHS resistance, more than what is caused or associated with the *HvMKK3* allele. Currently there is an increasing demand for barleys to be grown in non-traditional regions of the USA, many of which see high humidity and precipitation during harvest season. Successful expansion into these regions necessitates robust PHS resistance without reducing the quality malt produced. These results demonstrate combining substantial PHS protection and high malt quality in same variety is possible, even when malting at 48 days post PM. Discussions with maltsters indicate that about two months post-harvest maturity, about 70 days post PM, represents the earliest most malting operations can receive grain from a particular barley crop. Lastly, the malting process itself, duration and placement of steep and air-rests, and germination time allowed can affect rank order of malting quality characteristics and malting quality values (Brookes et al. 1976; Turner et al. 2019). In regions where the primary target customers are craft malting operations, lines unsuitable for large scale adjunct malting and brewing operations may perform well within smaller more flexible craft malting facilities (Brouwer et al. 2016). This is advantageous for the production of craft malts, as these facilities can be more flexible in the production of a malt that can be grown locally, successfully, without large risk of PHS.

### Further research

Expanding upon this work using a larger germplasm base (i.e., a diversity panel, or other more diverse set) would be useful. Correlations may be different in different sets of germplasm, environments and even within the germplasm from which these cultivars originated. Representatives of European, Canadian, and western US lines from North Dakota State University and Montana State University showed significant correlations of the malting quality traits to germination. Exact appraisal of the usefulness and estimated cost of using GI to select for both good malting quality and PHS resistance will need to be further examined in detail given the correlations and estimated cost of phenotyping each trait. Repeating this work under different steeping regimes may also be valuable. The S/T values obtained under the steeping regime from Schmitt and Budde, (2011) point to the production of extremely well or over modified malt. Gene knock-out experiments surrounding the *HvMKK3* and the resulting effect on malting quality would be informative. Further, the use of gene editing to introduce new alleles with intermediate phenotypes may have profound effects on combining good adjunct malting quality with PHS resistance following Hisano et al. (2022) who used Cas9-induced targeted mutagenesis on *HvAlaAT* and *HvMKK3* to generate mutants at each to work toward design of beneficial dormancy phenotypes for malting barley.

### General cautions to other breeding efforts

Breeding programs exist to improve crop genetics for increased production efficiency, whether that be by increasing the yield, quality, or any other number of relevant traits. Malting barley may serve as just one cautionary example that directly breeding for a primary trait may have undesirable consequences for other traits. We found that selection for high adjunct malt quality likely led to higher PHS susceptibility. This limits the adaptability of the bred varieties to new environments and increases the risk to malting barley producers. Any breeding undertaking should be carefully examined for unintended effects that selection for a target trait may have, weigh the cost and benefits of each effect, and modify the breeding program according to those conclusions. Most prominently, this may include incorporating balancing selection for the target and correlated traits so as to advance the target trait while minimizing the negative effect on the correlated traits.

## Conclusion

Germination traits, specifically GI at TP1, a proxy for PHS resistance, are significantly correlated across and within alleles of *HvMKK3* to malting quality traits, especially AA, FAN, SP, and S/T. Given these correlations, germination traits could be used in selection for potentially good malting candidates and PHS resistance, thus saving time and money on costly phenotyping. The Canadian style adjunct malting profile is associated with PHS susceptibility because the MKK_N*_ allele is also associated with malting quality traits. There is strong evidence this association is causative. PHS resistance comes with a cost, namely lower average FAN, AA, SP, and S/T trait values and a decreased probability of achieving an adjunct style malting quality trait profile. Here we provide evidence that selection of a primary trait can have either a desired or adverse effect on market value depending on the target market. All-malt target malting profiles are compatible with relatively high levels of PHS resistance and PHS resistant lines can be malted successfully even with relatively short periods of after-ripening. However, given that PHS resistance is unfavorably correlated with malting quality traits desired by the adjunct style malt brewers, constant care must be taken within active breeding programs during selection for adjunct malting quality to prevent PHS susceptibility.

## Supplementary Information

Below is the link to the electronic supplementary material.

Supplementary file1 (DOCX 183 KB)

## Data Availability

All data and analysis scripts will be made available in a GitHub repository.

## Abbreviations

AA: Alpha amylase activity
BLUEs: Best linear unbiased estimators
BG: Beta-glucan content
C0: Base population cycle
C1G: Cycle 1 genotypically selected lines
C2G: Cycle 2 genotypically selected lines
C1P: Cycle 1 phenotypically selected lines
CCRU_adj: Cereal crop research unit adjunct malting quality score
CCRU_allMalt: Cereal crop research unit all-malt malting quality score
DP: Diastatic power
FAN: Free amino nitrogen
GE: Germination energy (percentage)
GI: Germination index (rate)
GWA: Genome wide association
ME: Malt extract
MKK3_N*_: Highly non-dormant *HvMKK3* allele
MKK3_N_: Non-dormant *HvMKK3* allele
MKK3_D_: Dormant *HvMKK3* allele
MP: Malt protein
MTA: Marker trait association
PHS: Preharvest sprouting
PM: Physiological maturity
QTL: Quantitative trait loci
SP: Soluble protein
S/T: Soluble over total protein
TP1–7: Timepoints 1–7
