# Supplementary material for: Malting quality and preharvest sprouting traits are genetically correlated in spring malting barley
Source: Theor Appl Genet. 2023 Mar 13;136(3):59. doi: 10.1007/s00122-023-04257-6 (PMC10011292; doi:10.1007/s00122-023-04257-6)
Supplement: Supplementary file 1 — Supplementary file1 (DOCX 183 KB) [file 122_2023_4257_MOESM1_ESM.docx]

Supplemental Figure 1: Population creation and structure within the lines used in this study. AAC Synergy was crossed to 7 other parents to create the base population. Random selections from the base became C0. Phenotypic or genotypic selections were performed to create the subsequent cycles.
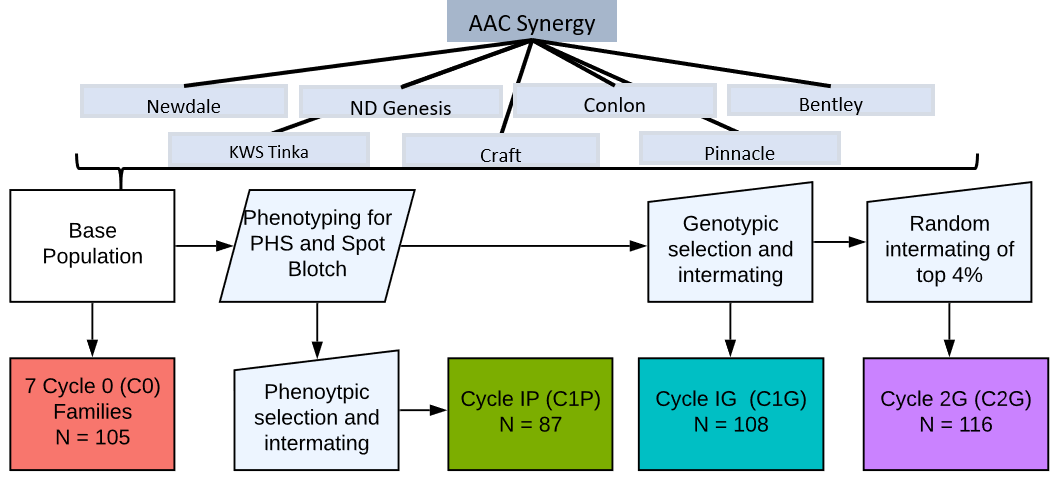


Supplemental Table 1: CCRU_Adj and CCRU_allMalt scoring parameters from the USDA Cereal Crops Research Unit in Madison, WI.

|  | 2-rowed Adjunct  CCRU_Adj | | 2-rowed All Malt  CCRU_allMalt | |
| --- | --- | --- | --- | --- |
| Quality parameter | condition | score | condition | score |
| Malt Extract (ME) | > 81.0 | 10 | > 81.5 | 10 |
| (% db) | 79.4-81.0 | 7 | 80.0-81.0 | 5 |
|  | 78.0-79.4 | 4 | 79.5-80.0 | 3 |
|  | <78.0 | 0 | <79.5 | 0 |
| Barley Protein (MP) | > 13.5 | 0 | > 11.5 | 0 |
| (% db) | 13.0-13.5 | 5 | 10.5-11.5 | 5 |
|  | 11.0-13.0 | 10 | 9.8-10.5 | 10 |
|  | < 11.0 | 5 | < 9.8 | 5 |
| Soluble Protein (SP) | > 6.0 | 0 | > 4.7 | 0 |
| (% db) | 5.6-6.0 | 3 | 4.5-4.7 | 3 |
|  | 4.8-5.6 | 7 | 4.1-4.5 | 7 |
|  | 4.0-4.8 | 3 | 3.9-4.1 | 3 |
|  | < 4.0 | 0 | < 3.9 | 0 |
| Soluble/Total | >47 | 0 | >45 | 0 |
| Protein, (S/T) % db | 40-47 | 5 | 43-45 | 3 |
|  | < 40 | 0 | 39-43 | 5 |
|  |  |  | <39 | 3 |
| Diastatic | >120 | 7 | >120 | 0 |
| Power, ° ASBC (DP) | 100-120 | 4 | 115-120 | 3 |
|  | < 100 | 0 | 100-115 | 7 |
|  |  |  | 95-100 | 3 |
|  |  |  | <95 | 0 |
| Alpha-amylase (AA) | >50 | 7 | >75 | 2 |
| (20° DU) | 40-50 | 4 | 65-75 | 5 |
|  | < 40 | 0 | < 65 | 0 |
| Beta-glucan (BG) | < 100 | 7 | < 100 | 7 |
| (ppm) | 100-150 | 3 | 100-150 | 3 |
|  | > 150 | 0 | > 150 | 0 |
| Free Amino Nitrogen (FAN)ppm | >210 | 5 | >200 | 0 |
|  | 180 - 210 | 3 | 180 - 200 | 3 |
|  | < 180 | 0 | 150-180 | 7 |
|  |  |  | 130-150 | 3 |
|  |  |  | <130 | 0 |
|  |  | 58 max |  | 58 max |

Supplemental Table 2: Model summary for genome-wide association (GWA) analysis. BLUE model indicates which model BLUEs were extracted from for GWA

| Year | Timepoint | Description | BLUEs model |
| --- | --- | --- | --- |
| 2019 | TP6 | per timepoint per year GWA | 4 |
| 2020 | TP4 | per timepoint per year GWA | 3 |
| 2020 | TP6 | per timepoint per year GWA | 3 |
| 2020 | TP4/TP6 | per year, treating timepoints as replicates | 4 |
| 2021 | TP4 | per timepoint per year GWA | 3 |
| 2021 | TP6 | per timepoint per year GWA | 3 |
| 2021 | TP4/TP6 | per year, treating timepoints as replicates | 4 |
| 2020/2021 | TP4 | over years, per timepoint | 5 |
| 2020/2021 | TP6 | over years, per timepoint | 5 |
| 2019/2020/2021 | TP6 | Over years TP6 malting timepoint | 5 |
| 2020/2021 | TP4/TP6 | over years, treating timepoints as replicates | 8 |
| Combined (2019/2020/2021) | TP4/TP6 | over years, treating timepoints as replicates | 8 |

Supplemental Table 3: Trait technical heritability within year and timepoint (TP),means, minimums, maximums, and standard deviations (sd).

| **year** | **TP** | **trait** | **TechH2** | **mean** | **sd** | **min** | **max** |
| --- | --- | --- | --- | --- | --- | --- | --- |
| 2019 | TP6 | AA | 0.88 | 72.12 | 14.50 | 32.47 | 130.65 |
| 2020 | TP4 | AA | 0.77 | 90.91 | 12.46 | 49.61 | 156.63 |
| 2020 | TP6 | AA | 0.84 | 88.10 | 11.67 | 52.00 | 130.17 |
| 2021 | TP4 | AA | 0.93 | 85.31 | 14.35 | 47.83 | 144.82 |
| 2021 | TP6 | AA | 0.93 | 93.27 | 15.05 | 41.71 | 141.68 |
| 2019 | TP6 | BG | 0.99 | 232.89 | 206.26 | 0.00 | 1440.89 |
| 2020 | TP4 | BG | 0.84 | 74.71 | 57.76 | 0.00 | 402.32 |
| 2020 | TP6 | BG | 0.82 | 84.16 | 65.32 | 0.00 | 521.32 |
| 2021 | TP4 | BG | 0.98 | 140.68 | 141.41 | 0.00 | 1164.42 |
| 2021 | TP6 | BG | 0.94 | 93.58 | 61.92 | 0.00 | 433.00 |
| 2019 | TP6 | DP | 0.83 | 104.29 | 27.09 | 28.30 | 304.09 |
| 2020 | TP4 | DP | 0.82 | 113.64 | 25.40 | 47.53 | 238.42 |
| 2020 | TP6 | DP | 0.87 | 113.46 | 27.37 | 23.06 | 209.16 |
| 2021 | TP4 | DP | 0.92 | 117.17 | 25.48 | 54.01 | 232.69 |
| 2021 | TP6 | DP | 0.87 | 117.70 | 22.04 | 42.79 | 187.09 |
| 2019 | TP6 | FAN | 0.84 | 193.05 | 33.53 | 59.00 | 354.96 |
| 2020 | TP4 | FAN | 0.82 | 275.94 | 48.45 | 115.61 | 462.70 |
| 2020 | TP6 | FAN | 0.73 | 282.77 | 44.66 | 170.64 | 445.70 |
| 2021 | TP4 | FAN | 0.84 | 215.59 | 39.75 | 116.64 | 342.96 |
| 2021 | TP6 | FAN | 0.88 | 222.15 | 35.00 | 123.83 | 398.40 |
| 2019 | TP6 | ME | 0.96 | 82.42 | 1.62 | 77.93 | 87.36 |
| 2020 | TP4 | ME | 0.98 | 82.40 | 1.33 | 77.00 | 85.82 |
| 2020 | TP6 | ME | 0.98 | 82.09 | 1.21 | 77.57 | 86.39 |
| 2021 | TP4 | ME | 0.98 | 82.82 | 1.10 | 79.32 | 85.62 |
| 2021 | TP6 | ME | 0.98 | 83.25 | 1.07 | 79.08 | 86.04 |
| 2019 | TP6 | MP |  | 9.47 | 0.96 | 7.36 | 13.55 |
| 2020 | TP6 | MP |  | 9.97 | 1.01 | 7.31 | 13.09 |
| 2019 | TP6 | SP | 0.98 | 4.98 | 0.56 | 3.43 | 6.79 |
| 2020 | TP4 | SP | 0.98 | 5.75 | 0.63 | 3.44 | 8.03 |
| 2020 | TP6 | SP | 0.94 | 6.01 | 0.63 | 3.88 | 7.85 |
| 2021 | TP4 | SP | 0.97 | 4.60 | 0.59 | 3.29 | 6.78 |
| 2021 | TP6 | SP | 0.97 | 4.43 | 0.51 | 3.09 | 6.79 |
| 2019 | TP6 | ST | 0.97 | 0.53 | 0.04 | 0.42 | 0.68 |
| 2020 | TP4 | ST | 0.99 | 0.57 | 0.08 | 0.35 | 0.85 |
| 2020 | TP6 | ST | 0.93 | 0.60 | 0.05 | 0.32 | 0.74 |
| 2021 | TP4 | ST | 0.96 | 0.51 | 0.05 | 0.37 | 0.70 |
| 2021 | TP6 | ST | 0.93 | 0.50 | 0.04 | 0.39 | 0.63 |

Supplemental Figure 2: Malting quality trait values and distributions per year and time point.


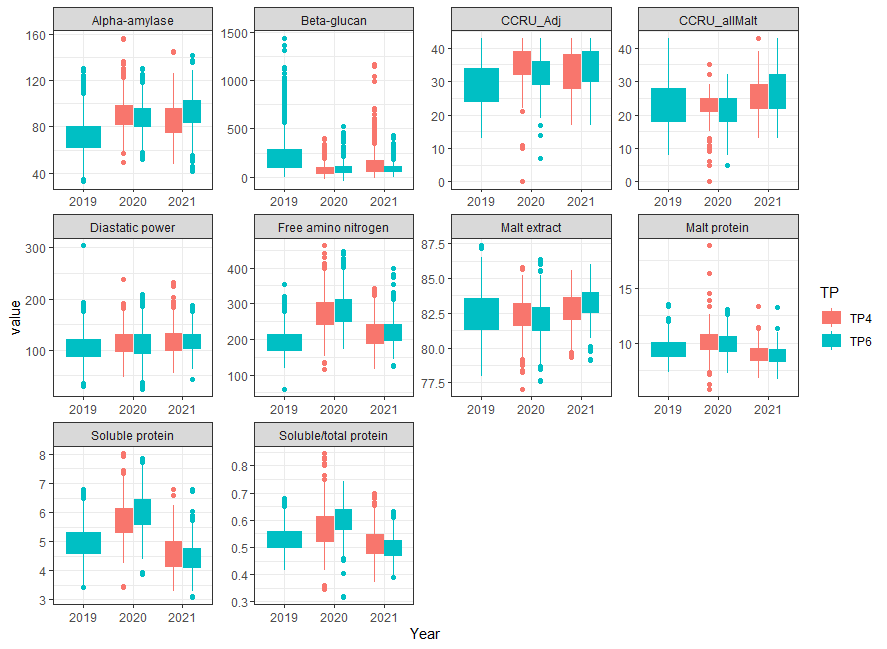


| **Year** | **trait** | **YearH^2^** | **CullisH^2^** | **σ^2^_g_** | **σ^2^_g:TP_** | **σ^2^_e_** | **σ^2^ _Tradition_** | **σ^2^_Δg:TP_** | **ratio1** | **ratio2** |
| --- | --- | --- | --- | --- | --- | --- | --- | --- | --- | --- |
| 2020 | AA | 0.413 | 0.786 | 59.397 | 54.419 | 29.98 | 54.094 | 30.305 | 0.557 | 0.51 |
| 2020 | BG | 0.333 | 0.747 | 1290.443 | 1618.689 | 968.045 | 4746.094 | -2159.36 | -1.334 | -1.673 |
| 2020 | DP | 0.525 | 0.836 | 382.808 | 230.186 | 115.97 | 275.322 | 70.834 | 0.308 | 0.185 |
| 2020 | FAN | 0.549 | 0.866 | 1180.013 | 426.228 | 544.164 | 774.722 | 195.67 | 0.459 | 0.166 |
| 2020 | ME | 0.339 | 0.721 | 0.548 | 0.96 | 0.11 | 0.447 | 0.623 | 0.649 | 1.136 |
| 2020 | MP | 0.337 | 0.716 | 0.451 | 0.449 | 0.436 |  |  |  |  |
| 2020 | SP | 0.665 | 0.881 | 0.272 | 0.106 | 0.031 | 0.076 | 0.061 | 0.578 | 0.225 |
| 2020 | ST | 0.207 | 0.645 | 0.001 | 0.003 | 0 |  |  |  |  |
| 2021 | AA | 0.448 | 0.82 | 99.137 | 105.611 | 16.305 | 54.094 | 67.822 | 0.642 | 0.684 |
| 2021 | BG | 0.306 | 0.737 | 4236.16 | 9252.051 | 358.569 | 4746.094 | 4864.527 | 0.526 | 1.148 |
| 2021 | DP | 0.501 | 0.85 | 288.537 | 223.265 | 64.314 | 275.322 | 12.257 | 0.055 | 0.042 |
| 2021 | FAN | 0.471 | 0.845 | 677.706 | 532.563 | 227.926 | 774.722 | -14.232 | -0.027 | -0.021 |
| 2021 | ME | 0.544 | 0.856 | 0.651 | 0.511 | 0.034 | 0.447 | 0.099 | 0.193 | 0.151 |
| 2021 | MP | 0.995 | 0.999 | 0.786 | 0 | 0.004 |  |  |  |  |
| 2021 | SP | 0.693 | 0.914 | 0.21 | 0.082 | 0.012 | 0.076 | 0.017 | 0.211 | 0.082 |
| 2021 | ST | 0.51 | 0.846 | 0.001 | 0.001 | 0 |  |  |  |  |

Supplemental Table 4: Plot level error ( σ_pltE_^2^ ) and σ_Δg:TP_^2^ estimation. ratio1 = σ^2^**_Δg:TP_ /** σ^2^**_g:TP_** a value <0.5 indicates the majority of line:TP variance was due to error. Ratio2 = σ^2^**_Δg:TP_ /** σ^2^**_g_** a value < 0.5 indicates lower relative importance or the line:TP deviations as compared to the main taxa effect.

Supplemental Table 5: Year basis heritability for all malting quality traits treating malting timepoint a fixed effect in the model.

| **Year** | **trait** | **H2** | **CullisH2** | **σ^2^_g_** | **σ^2^_g:TP_** | **σ^2^_e_** | **ratio2** |
| --- | --- | --- | --- | --- | --- | --- | --- |
| Combined | AA | 0.523 | 0.843 | 63.876 | 27.026 | 93.875 | 0.423 |
| Combined | BG | 0.373 | 0.767 | 4189.747 | 3823.8 | 9657.101 | 0.913 |
| Combined | CCRU_Adj | 0.305 | 0.664 | 4.386 | 0 | 30.056 | 0 |
| Combined | CCRU_allMalt | 0.172 | 0.597 | 2.229 | 0 | 32.086 | 0 |
| Combined | DP | 0.588 | 0.875 | 270.476 | 74.041 | 345.75 | 0.274 |
| Combined | FAN | 0.589 | 0.876 | 636.154 | 155.357 | 868.22 | 0.244 |
| Combined | ME | 0.494 | 0.816 | 0.548 | 0.221 | 1.019 | 0.403 |
| Combined | MP | 0.678 | 0.839 | 0.451 | 0 | 0.643 | 0 |
| Combined | SP | 0.734 | 0.916 | 0.199 | 0.028 | 0.133 | 0.139 |
| Combined | ST | 0.39 | 0.759 | 0.001 | 0.001 | 0.001 | 1.02 |

Supplemental Table 6: Significant MTA for the GWA on the malting quality traits. MAF, Minor allele frequency; N_obs_ number of observations. Year and TP give the years and the timepoints that were compiled within a linear model to estimate the BLUEs input into the GWA. Positions are relative to the Morex V2 reference genome.

| **year** | **TP** | **trait** | **SNP** | **Chromosome** | **Position** | **P.value** | **MAF** | **N_obs_** |
| --- | --- | --- | --- | --- | --- | --- | --- | --- |
| Combined | TP4/TP6 | AA | JHI-Hv50k-2016-415600 | 6 | 526130710 | 1.32E-08 | 0.15 | 422 |
| Combined | TP4/TP6 | AA | MKK3_E165Q | 5 | 596732030 | 1.82E-09 | 0.21 | 422 |
| Combined | TP4/TP6 | BG | JHI-Hv50k-2016-388539 | 6 | 69601728 | 0.00000057 | 0.28 | 422 |
| Combined | TP4/TP6 | DP | JHI-Hv50k-2016-386349 | 6 | 47982141 | 0.000000335 | 0.3 | 422 |
| Combined | TP4/TP6 | FAN | JHI-Hv50k-2016-385463 | 6 | 42618343 | 0.0000013 | 0.29 | 422 |
| Combined | TP4/TP6 | FAN | MKK3_E165Q | 5 | 596732030 | 5.87E-22 | 0.21 | 422 |
| Combined | TP4/TP6 | FAN | SCRI_RS_4520 | 7 | 610967042 | 4.14E-08 | 0.07 | 422 |
| Combined | TP4/TP6 | MP | JHI-Hv50k-2016-385463 | 6 | 42618343 | 4.18E-11 | 0.29 | 422 |
| Combined | TP4/TP6 | SP | JHI-Hv50k-2016-365992 | 5 | 594326624 | 9.06E-16 | 0.31 | 422 |
| Combined | TP4/TP6 | SP | JHI-Hv50k-2016-385463 | 6 | 42618343 | 0.000000696 | 0.29 | 422 |
| Combined | TP4/TP6 | SP | JHI-Hv50k-2016-48870 | 1 | 497105966 | 0.0000321 | 0.15 | 422 |
| Combined | TP4/TP6 | SP | JHI-Hv50k-2016-49136 | 1 | 499170798 | 0.0000246 | 0.15 | 422 |
| Combined | TP4/TP6 | ST | JHI-Hv50k-2016-365992 | 5 | 594326624 | 0.000000125 | 0.31 | 422 |
| 2021 | TP4 | AA | JHI-Hv50k-2016-146702 | 2 | 671664303 | 0.00000208 | 0.35 | 235 |
| 2021 | TP4 | AA | MKK3_E165Q | 5 | 596732030 | 6.62E-14 | 0.23 | 235 |
| 2021 | TP4 | BG | JHI-Hv50k-2016-324079 | 5 | 516095610 | 0.0000401 | 0.09 | 235 |
| 2021 | TP4 | BG | JHI-Hv50k-2016-324259 | 5 | 516763212 | 0.0000401 | 0.09 | 235 |
| 2021 | TP4 | BG | JHI-Hv50k-2016-324279 | 5 | 516791825 | 0.0000401 | 0.09 | 235 |
| 2021 | TP4 | BG | JHI-Hv50k-2016-324331 | 5 | 516987801 | 0.0000401 | 0.09 | 235 |
| 2021 | TP4 | BG | JHI-Hv50k-2016-324352 | 5 | 516983587 | 0.0000401 | 0.09 | 235 |
| 2021 | TP4 | BG | JHI-Hv50k-2016-324426 | 5 | 516935881 | 0.0000409 | 0.09 | 235 |
| 2021 | TP4 | BG | JHI-Hv50k-2016-324435 | 5 | 516935567 | 0.0000401 | 0.09 | 235 |
| 2021 | TP4 | BG | JHI-Hv50k-2016-324546 | 5 | 517420548 | 0.0000401 | 0.09 | 235 |
| 2021 | TP4 | BG | JHI-Hv50k-2016-324622 | 5 | 517411628 | 0.0000401 | 0.09 | 235 |
| 2021 | TP4 | BG | JHI-Hv50k-2016-324709 | 5 | 517400601 | 0.0000401 | 0.09 | 235 |
| 2021 | TP4 | BG | JHI-Hv50k-2016-324732 | 5 | 517398726 | 0.0000238 | 0.1 | 235 |
| 2021 | TP4 | BG | JHI-Hv50k-2016-324773 | 5 | 517346418 | 0.0000413 | 0.09 | 235 |
| 2021 | TP4 | BG | JHI-Hv50k-2016-324774 | 5 | 517346327 | 0.0000256 | 0.1 | 235 |
| 2021 | TP4 | BG | JHI-Hv50k-2016-324786 | 5 | 517601596 | 0.0000401 | 0.09 | 235 |
| 2021 | TP4 | BG | JHI-Hv50k-2016-324907 | 5 | 517572975 | 0.000025 | 0.1 | 235 |
| 2021 | TP4 | BG | SCRI_RS_224501 | 5 | 517398619 | 0.000032 | 0.1 | 235 |
| 2021 | TP4 | DP | JHI-Hv50k-2016-275749 | 4 | 622111029 | 0.0000282 | 0.19 | 235 |
| 2021 | TP4 | FAN | JHI-Hv50k-2016-365992 | 5 | 594326624 | 1.27E-12 | 0.33 | 235 |
| 2021 | TP4 | SP | JHI-Hv50k-2016-503859 | 7 | 603982673 | 0.0000262 | 0.1 | 235 |
| 2021 | TP4 | SP | JHI-Hv50k-2016-507120 | 7 | 610777620 | 0.0000141 | 0.08 | 235 |
| 2021 | TP4 | SP | MKK3_E165Q | 5 | 596732030 | 0.000000548 | 0.23 | 235 |
| 2021 | TP4 | SP | SCRI_RS_994 | 7 | 610967943 | 0.0000141 | 0.08 | 235 |
| 2021 | TP4/TP6 | AA | JHI-Hv50k-2016-415494 | 6 | 525689623 | 0.00000289 | 0.14 | 235 |
| 2021 | TP4/TP6 | AA | MKK3_E165Q | 5 | 596732030 | 4.77E-10 | 0.23 | 235 |
| 2021 | TP4/TP6 | DP | JHI-Hv50k-2016-275749 | 4 | 622111029 | 0.0000101 | 0.19 | 235 |
| 2021 | TP4/TP6 | FAN | JHI-Hv50k-2016-131168 | 2 | 646227051 | 0.00000386 | 0.11 | 235 |
| 2021 | TP4/TP6 | FAN | MKK3_E165Q | 5 | 596732030 | 1.51E-18 | 0.23 | 235 |
| 2021 | TP4/TP6 | SP | JHI-Hv50k-2016-503859 | 7 | 603982673 | 0.0000122 | 0.1 | 235 |
| 2021 | TP4/TP6 | SP | JHI-Hv50k-2016-507120 | 7 | 610777620 | 0.000015 | 0.08 | 235 |
| 2021 | TP4/TP6 | SP | MKK3_E165Q | 5 | 596732030 | 0.000000152 | 0.23 | 235 |
| 2021 | TP4/TP6 | SP | SCRI_RS_994 | 7 | 610967943 | 0.000015 | 0.08 | 235 |
| 2021 | TP6 | AA | JHI-Hv50k-2016-365992 | 5 | 594326624 | 0.0000287 | 0.33 | 235 |
| 2021 | TP6 | DP | JHI-Hv50k-2016-113922 | 2 | 611118129 | 0.0000489 | 0.12 | 235 |
| 2021 | TP6 | FAN | JHI-Hv50k-2016-507120 | 7 | 610777620 | 0.0000325 | 0.08 | 235 |
| 2021 | TP6 | FAN | MKK3_E165Q | 5 | 596732030 | 3.65E-15 | 0.23 | 235 |
| 2021 | TP6 | FAN | SCRI_RS_4520 | 7 | 610967042 | 0.0000349 | 0.07 | 235 |
| 2021 | TP6 | FAN | SCRI_RS_994 | 7 | 610967943 | 0.0000325 | 0.08 | 235 |
| 2021 | TP6 | SP | SCRI_RS_99344 | 5 | 594204409 | 0.000000131 | 0.24 | 235 |
| 2020/2021 | TP4 | AA | MKK3_E165Q | 5 | 596732030 | 0.000000906 | 0.22 | 236 |
| 2020/2021 | TP4 | BG | JHI-Hv50k-2016-319453 | 5 | 501613478 | 0.000000666 | 0.1 | 237 |
| 2020/2021 | TP4 | CCRU_Adj | JHI-Hv50k-2016-319874 | 5 | 502767380 | 0.000000513 | 0.1 | 236 |
| 2020/2021 | TP4 | CCRU_allMalt | JHI-Hv50k-2016-508688 | 7 | 614933750 | 0.00000102 | 0.16 | 236 |
| 2020/2021 | TP4 | FAN | SCRI_RS_99344 | 5 | 594204409 | 1.58E-10 | 0.23 | 237 |
| 2020/2021 | TP4 | ME | JHI-Hv50k-2016-106063 | 2 | 575919841 | 0.0000389 | 0.21 | 236 |
| 2020/2021 | TP4 | SP | JHI-Hv50k-2016-365992 | 5 | 594326624 | 0.0000011 | 0.33 | 236 |
| 2020/2021 | TP4 | SP | SCRI_RS_13960 | 5 | 505259545 | 0.0000181 | 0.32 | 236 |
| 2020/2021 | TP4 | SP | SCRI_RS_157334 | 5 | 505757752 | 0.0000209 | 0.32 | 236 |
| 2020/2021 | TP4/TP6 | AA | JHI-Hv50k-2016-415658 | 6 | 526387284 | 9.58E-10 | 0.14 | 421 |
| 2020/2021 | TP4/TP6 | AA | MKK3_E165Q | 5 | 596732030 | 0.000000109 | 0.21 | 421 |
| 2020/2021 | TP4/TP6 | CCRU_Adj | JHI-Hv50k-2016-415610 | 6 | 526079023 | 0.0000303 | 0.14 | 421 |
| 2020/2021 | TP4/TP6 | CCRU_allMalt | JHI-Hv50k-2016-222407 | 3 | 618534459 | 0.0000236 | 0.14 | 421 |
| 2020/2021 | TP4/TP6 | CCRU_allMalt | JHI-Hv50k-2016-222417 | 3 | 618537703 | 0.0000236 | 0.14 | 421 |
| 2020/2021 | TP4/TP6 | DP | JHI-Hv50k-2016-415494 | 6 | 525689623 | 0.000024 | 0.15 | 421 |
| 2020/2021 | TP4/TP6 | DP | JHI-Hv50k-2016-415600 | 6 | 526130710 | 0.0000363 | 0.15 | 421 |
| 2020/2021 | TP4/TP6 | DP | JHI-Hv50k-2016-415610 | 6 | 526079023 | 0.0000235 | 0.14 | 421 |
| 2020/2021 | TP4/TP6 | DP | JHI-Hv50k-2016-415658 | 6 | 526387284 | 0.0000434 | 0.14 | 421 |
| 2020/2021 | TP4/TP6 | FAN | JHI-Hv50k-2016-506938 | 7 | 610478589 | 0.0000305 | 0.08 | 420 |
| 2020/2021 | TP4/TP6 | FAN | JHI-Hv50k-2016-507120 | 7 | 610777620 | 0.0000187 | 0.08 | 420 |
| 2020/2021 | TP4/TP6 | FAN | MKK3_E165Q | 5 | 596732030 | 1.45E-12 | 0.22 | 420 |
| 2020/2021 | TP4/TP6 | FAN | SCRI_RS_994 | 7 | 610967943 | 0.0000187 | 0.08 | 420 |
| 2020/2021 | TP4/TP6 | SP | SCRI_RS_99344 | 5 | 594204409 | 2E-12 | 0.22 | 420 |
| 2020/2021 | TP6 | AA | JHI-Hv50k-2016-415658 | 6 | 526387284 | 2.58E-09 | 0.14 | 421 |
| 2020/2021 | TP6 | AA | MKK3_E165Q | 5 | 596732030 | 0.00000307 | 0.21 | 421 |
| 2020/2021 | TP6 | FAN | JHI-Hv50k-2016-230574 | 4 | 13628037 | 0.0000434 | 0.2 | 420 |
| 2020/2021 | TP6 | FAN | JHI-Hv50k-2016-230622 | 4 | 13890108 | 0.0000434 | 0.2 | 420 |
| 2020/2021 | TP6 | FAN | JHI-Hv50k-2016-230631 | 4 | 13715530 | 0.0000434 | 0.2 | 420 |
| 2020/2021 | TP6 | FAN | JHI-Hv50k-2016-230656 | 4 | 13737568 | 0.0000434 | 0.2 | 420 |
| 2020/2021 | TP6 | FAN | JHI-Hv50k-2016-230714 | 4 | 13973109 | 0.0000434 | 0.2 | 420 |
| 2020/2021 | TP6 | FAN | JHI-Hv50k-2016-506938 | 7 | 610478589 | 0.00000874 | 0.08 | 420 |
| 2020/2021 | TP6 | FAN | JHI-Hv50k-2016-507120 | 7 | 610777620 | 0.00000598 | 0.08 | 420 |
| 2020/2021 | TP6 | FAN | MKK3_E165Q | 5 | 596732030 | 6.96E-14 | 0.22 | 420 |
| 2020/2021 | TP6 | FAN | SCRI_RS_127657 | 4 | 13883092 | 0.0000434 | 0.2 | 420 |
| 2020/2021 | TP6 | FAN | SCRI_RS_154327 | 7 | 610940992 | 0.0000299 | 0.09 | 420 |
| 2020/2021 | TP6 | FAN | SCRI_RS_155132 | 7 | 610446184 | 0.0000299 | 0.09 | 420 |
| 2020/2021 | TP6 | FAN | SCRI_RS_4520 | 7 | 610967042 | 0.000025 | 0.07 | 420 |
| 2020/2021 | TP6 | FAN | SCRI_RS_98443 | 4 | 14583570 | 0.0000281 | 0.15 | 420 |
| 2020/2021 | TP6 | FAN | SCRI_RS_994 | 7 | 610967943 | 0.00000598 | 0.08 | 420 |
| 2020/2021 | TP6 | SP | SCRI_RS_99344 | 5 | 594204409 | 1.89E-14 | 0.22 | 420 |
| 2020 | TP4 | BG | JHI-Hv50k-2016-319453 | 5 | 501613478 | 0.00000121 | 0.09 | 231 |
| 2020 | TP4 | FAN | JHI-Hv50k-2016-194935 | 3 | 526205490 | 0.00000813 | 0.14 | 231 |
| 2020 | TP4 | FAN | SCRI_RS_151407 | 5 | 593317767 | 0.0000104 | 0.28 | 231 |
| 2020 | TP4 | FAN | SCRI_RS_99344 | 5 | 594204409 | 0.0000239 | 0.23 | 231 |
| 2020 | TP4 | ME | BOPA1_ABC04861-2-1-334 | 2 | 579076167 | 0.00000677 | 0.21 | 229 |
| 2020 | TP4 | ME | JHI-Hv50k-2016-106063 | 2 | 575919841 | 0.0000246 | 0.21 | 229 |
| 2020 | TP4 | ME | JHI-Hv50k-2016-106085 | 2 | 576189421 | 0.0000203 | 0.22 | 229 |
| 2020 | TP4 | ME | JHI-Hv50k-2016-106100 | 2 | 576187598 | 0.0000104 | 0.21 | 229 |
| 2020 | TP4 | ME | JHI-Hv50k-2016-106163 | 2 | 576303991 | 0.0000104 | 0.21 | 229 |
| 2020 | TP4 | ME | JHI-Hv50k-2016-106171 | 2 | 576555711 | 0.0000244 | 0.22 | 229 |
| 2020 | TP4 | ME | JHI-Hv50k-2016-106192 | 2 | 576550833 | 0.0000104 | 0.21 | 229 |
| 2020 | TP4 | ME | JHI-Hv50k-2016-106211 | 2 | 576675329 | 0.0000104 | 0.21 | 229 |
| 2020 | TP4 | ME | JHI-Hv50k-2016-106221 | 2 | 576671807 | 0.00000954 | 0.22 | 229 |
| 2020 | TP4 | ME | JHI-Hv50k-2016-106229 | 2 | 577014537 | 0.0000487 | 0.2 | 229 |
| 2020 | TP4 | ME | JHI-Hv50k-2016-106243 | 2 | 577100660 | 0.00000806 | 0.21 | 229 |
| 2020 | TP4 | ME | JHI-Hv50k-2016-106268 | 2 | 577289059 | 0.00000881 | 0.21 | 229 |
| 2020 | TP4 | ME | JHI-Hv50k-2016-106282 | 2 | 577355021 | 0.00000881 | 0.21 | 229 |
| 2020 | TP4 | ME | JHI-Hv50k-2016-106306 | 2 | 577480276 | 0.0000376 | 0.21 | 229 |
| 2020 | TP4 | ME | JHI-Hv50k-2016-106330 | 2 | 577441561 | 0.00000881 | 0.21 | 229 |
| 2020 | TP4 | ME | JHI-Hv50k-2016-106353 | 2 | 577410937 | 0.000015 | 0.21 | 229 |
| 2020 | TP4 | ME | JHI-Hv50k-2016-106370 | 2 | 577512543 | 0.00000881 | 0.21 | 229 |
| 2020 | TP4 | ME | JHI-Hv50k-2016-106371 | 2 | 577512439 | 0.00000881 | 0.21 | 229 |
| 2020 | TP4 | ME | JHI-Hv50k-2016-106390 | 2 | 577502854 | 0.00000803 | 0.21 | 229 |
| 2020 | TP4 | ME | JHI-Hv50k-2016-106397 | 2 | 577653116 | 0.00000934 | 0.21 | 229 |
| 2020 | TP4 | ME | JHI-Hv50k-2016-106402 | 2 | 577740265 | 0.00000934 | 0.21 | 229 |
| 2020 | TP4 | ME | JHI-Hv50k-2016-106449 | 2 | 577877612 | 0.00000934 | 0.21 | 229 |
| 2020 | TP4 | ME | JHI-Hv50k-2016-106459 | 2 | 578064200 | 0.00000934 | 0.21 | 229 |
| 2020 | TP4 | ME | JHI-Hv50k-2016-106502 | 2 | 578052429 | 0.00000934 | 0.21 | 229 |
| 2020 | TP4 | ME | JHI-Hv50k-2016-106525 | 2 | 578123460 | 0.00000934 | 0.21 | 229 |
| 2020 | TP4 | ME | JHI-Hv50k-2016-106526 | 2 | 578123699 | 0.00000934 | 0.21 | 229 |
| 2020 | TP4 | ME | JHI-Hv50k-2016-106554 | 2 | 578277884 | 0.00000934 | 0.21 | 229 |
| 2020 | TP4 | ME | JHI-Hv50k-2016-106820 | 2 | 579356193 | 0.00000642 | 0.21 | 229 |
| 2020 | TP4 | ME | JHI-Hv50k-2016-106842 | 2 | 579377258 | 0.0000174 | 0.2 | 229 |
| 2020 | TP4 | ME | SCRI_RS_154398 | 2 | 577417224 | 0.00000881 | 0.21 | 229 |
| 2020 | TP4 | ME | SCRI_RS_4930 | 2 | 578888974 | 0.0000163 | 0.2 | 229 |
| 2020 | TP4 | MP | JHI-Hv50k-2016-385540 | 6 | 43219101 | 0.000000885 | 0.23 | 233 |
| 2020 | TP4 | SP | JHI-Hv50k-2016-365992 | 5 | 594326624 | 0.00000295 | 0.33 | 227 |
| 2020 | TP4/TP6 | AA | JHI-Hv50k-2016-415658 | 6 | 526387284 | 0.000000668 | 0.14 | 421 |
| 2020 | TP4/TP6 | DP | JHI-Hv50k-2016-385463 | 6 | 42618343 | 0.0000197 | 0.29 | 421 |
| 2020 | TP4/TP6 | DP | JHI-Hv50k-2016-415610 | 6 | 526079023 | 0.0000483 | 0.14 | 421 |
| 2020 | TP4/TP6 | FAN | SCRI_RS_99344 | 5 | 594204409 | 3.35E-10 | 0.22 | 420 |
| 2020 | TP4/TP6 | ME | BOPA1_ABC04861-2-1-334 | 2 | 579076167 | 0.0000236 | 0.21 | 420 |
| 2020 | TP4/TP6 | ME | JHI-Hv50k-2016-106397 | 2 | 577653116 | 0.0000247 | 0.21 | 420 |
| 2020 | TP4/TP6 | ME | JHI-Hv50k-2016-106402 | 2 | 577740265 | 0.0000247 | 0.21 | 420 |
| 2020 | TP4/TP6 | ME | JHI-Hv50k-2016-106449 | 2 | 577877612 | 0.0000247 | 0.21 | 420 |
| 2020 | TP4/TP6 | ME | JHI-Hv50k-2016-106459 | 2 | 578064200 | 0.0000247 | 0.21 | 420 |
| 2020 | TP4/TP6 | ME | JHI-Hv50k-2016-106502 | 2 | 578052429 | 0.0000247 | 0.21 | 420 |
| 2020 | TP4/TP6 | ME | JHI-Hv50k-2016-106525 | 2 | 578123460 | 0.0000247 | 0.21 | 420 |
| 2020 | TP4/TP6 | ME | JHI-Hv50k-2016-106526 | 2 | 578123699 | 0.0000247 | 0.21 | 420 |
| 2020 | TP4/TP6 | ME | JHI-Hv50k-2016-106554 | 2 | 578277884 | 0.0000244 | 0.21 | 420 |
| 2020 | TP4/TP6 | ME | JHI-Hv50k-2016-106820 | 2 | 579356193 | 0.0000182 | 0.21 | 420 |
| 2020 | TP4/TP6 | MP | JHI-Hv50k-2016-385463 | 6 | 42618343 | 3.4E-10 | 0.29 | 421 |
| 2020 | TP4/TP6 | SP | JHI-Hv50k-2016-385463 | 6 | 42618343 | 3.59E-08 | 0.28 | 420 |
| 2020 | TP4/TP6 | SP | SCRI_RS_99344 | 5 | 594204409 | 3.09E-13 | 0.22 | 420 |
| 2020 | TP6 | AA | JHI-Hv50k-2016-415610 | 6 | 526079023 | 0.0000012 | 0.14 | 421 |
| 2020 | TP6 | FAN | JHI-Hv50k-2016-506938 | 7 | 610478589 | 0.0000311 | 0.08 | 420 |
| 2020 | TP6 | FAN | JHI-Hv50k-2016-507120 | 7 | 610777620 | 0.0000288 | 0.08 | 420 |
| 2020 | TP6 | FAN | SCRI_RS_99344 | 5 | 594204409 | 4.32E-11 | 0.22 | 420 |
| 2020 | TP6 | FAN | SCRI_RS_994 | 7 | 610967943 | 0.0000288 | 0.08 | 420 |
| 2020 | TP6 | ME | JHI-Hv50k-2016-100999 | 2 | 546772860 | 0.0000499 | 0.13 | 420 |
| 2020 | TP6 | MP | JHI-Hv50k-2016-385463 | 6 | 42618343 | 0.000000001 | 0.29 | 421 |
| 2020 | TP6 | SP | JHI-Hv50k-2016-385463 | 6 | 42618343 | 2.4E-09 | 0.28 | 420 |
| 2020 | TP6 | SP | SCRI_RS_99344 | 5 | 594204409 | 2E-14 | 0.22 | 420 |
| 2020 | TP6 | S/T | SCRI_RS_99344 | 5 | 594204409 | 0.0000423 | 0.22 | 420 |
| 2019 | TP6 | AA | BOPA2_12_30658 | 6 | 49027068 | 0.00000022 | 0.28 | 366 |
| 2019 | TP6 | AA | BOPA2_12_31267 | 5 | 592906854 | 0.000000363 | 0.32 | 366 |
| 2019 | TP6 | AA | JHI-Hv50k-2016-455148 | 7 | 25961769 | 0.0000022 | 0.27 | 366 |
| 2019 | TP6 | BG | JHI-Hv50k-2016-387236 | 6 | 57403051 | 3.89E-08 | 0.26 | 366 |
| 2019 | TP6 | DP | BOPA2_12_30658 | 6 | 49027068 | 0.0000275 | 0.28 | 366 |
| 2019 | TP6 | DP | JHI-Hv50k-2016-385463 | 6 | 42618343 | 0.0000408 | 0.26 | 366 |
| 2019 | TP6 | DP | JHI-Hv50k-2016-386215 | 6 | 46682009 | 0.0000438 | 0.28 | 366 |
| 2019 | TP6 | DP | JHI-Hv50k-2016-386349 | 6 | 47982141 | 0.0000438 | 0.28 | 366 |
| 2019 | TP6 | DP | JHI-Hv50k-2016-386406 | 6 | 48076092 | 0.0000438 | 0.28 | 366 |
| 2019 | TP6 | DP | JHI-Hv50k-2016-386452 | 6 | 48443762 | 0.0000438 | 0.28 | 366 |
| 2019 | TP6 | DP | JHI-Hv50k-2016-386678 | 6 | 49498317 | 0.0000438 | 0.28 | 366 |
| 2019 | TP6 | DP | JHI-Hv50k-2016-386727 | 6 | 50086518 | 0.0000438 | 0.28 | 366 |
| 2019 | TP6 | DP | JHI-Hv50k-2016-386744 | 6 | 50542675 | 0.0000412 | 0.28 | 366 |
| 2019 | TP6 | DP | JHI-Hv50k-2016-387236 | 6 | 57403051 | 0.0000428 | 0.26 | 366 |
| 2019 | TP6 | DP | SCRI_RS_168111 | 6 | 49811922 | 0.0000438 | 0.28 | 366 |
| 2019 | TP6 | DP | SCRI_RS_168455 | 6 | 50782567 | 0.0000438 | 0.28 | 366 |
| 2019 | TP6 | DP | SCRI_RS_196459 | 6 | 48797225 | 0.0000438 | 0.28 | 366 |
| 2019 | TP6 | FAN | MKK3_E165Q | 5 | 596732030 | 4.55E-12 | 0.22 | 366 |
| 2019 | TP6 | MP | JHI-Hv50k-2016-49136 | 1 | 499170798 | 0.0000469 | 0.15 | 363 |
| 2019 | TP6 | SP | JHI-Hv50k-2016-366380 | 5 | 595767289 | 3.16E-10 | 0.22 | 365 |
| 2019 | TP6 | S/T | JHI-Hv50k-2016-366380 | 5 | 595767289 | 0.000000131 | 0.22 | 361 |
| 19/20/21 | TP6 | AA | JHI-Hv50k-2016-415600 | 6 | 526130710 | 0.000000365 | 0.15 | 422 |
| 19/20/21 | TP6 | AA | SCRI_RS_120501 | 5 | 595207219 | 4.39E-08 | 0.22 | 422 |
| 19/20/21 | TP6 | BG | JHI-Hv50k-2016-387236 | 6 | 57403051 | 9.74E-08 | 0.28 | 422 |
| 19/20/21 | TP6 | DP | JHI-Hv50k-2016-386349 | 6 | 47982141 | 0.000000679 | 0.3 | 422 |
| 19/20/21 | TP6 | FAN | JHI-Hv50k-2016-385463 | 6 | 42618343 | 0.00000118 | 0.29 | 422 |
| 19/20/21 | TP6 | FAN | MKK3_E165Q | 5 | 596732030 | 1.77E-22 | 0.21 | 422 |
| 19/20/21 | TP6 | FAN | SCRI_RS_4520 | 7 | 610967042 | 3.55E-08 | 0.07 | 422 |
| 19/20/21 | TP6 | MP | BOPA1_1062-478 | 2 | 365198431 | 0.0000417 | 0.33 | 422 |
| 19/20/21 | TP6 | MP | BOPA1_2528-2111 | 2 | 342925311 | 0.0000444 | 0.33 | 422 |
| 19/20/21 | TP6 | MP | BOPA1_3355-605 | 2 | 459638235 | 0.0000444 | 0.33 | 422 |
| 19/20/21 | TP6 | MP | BOPA2_12_10035 | 2 | 414970641 | 0.0000444 | 0.33 | 422 |
| 19/20/21 | TP6 | MP | BOPA2_12_11504 | 2 | 465651590 | 0.0000444 | 0.33 | 422 |
| 19/20/21 | TP6 | MP | BOPA2_12_30561 | 2 | 313650588 | 0.0000319 | 0.33 | 422 |
| 19/20/21 | TP6 | MP | BOPA2_12_30582 | 2 | 257254188 | 0.0000444 | 0.33 | 422 |
| 19/20/21 | TP6 | MP | BOPA2_12_31175 | 2 | 280911757 | 0.0000417 | 0.33 | 422 |
| 19/20/21 | TP6 | MP | JHI-Hv50k-2016-385463 | 6 | 42618343 | 1.59E-10 | 0.29 | 422 |
| 19/20/21 | TP6 | MP | JHI-Hv50k-2016-49136 | 1 | 499170798 | 0.0000353 | 0.15 | 422 |
| 19/20/21 | TP6 | MP | JHI-Hv50k-2016-90385 | 2 | 362597415 | 0.0000444 | 0.33 | 422 |
| 19/20/21 | TP6 | MP | JHI-Hv50k-2016-91952 | 2 | 224081977 | 0.0000444 | 0.33 | 422 |
| 19/20/21 | TP6 | MP | JHI-Hv50k-2016-92134 | 2 | 261680302 | 0.0000444 | 0.33 | 422 |
| 19/20/21 | TP6 | MP | JHI-Hv50k-2016-92202 | 2 | 272501172 | 0.0000444 | 0.33 | 422 |
| 19/20/21 | TP6 | MP | JHI-Hv50k-2016-92456 | 2 | 322103293 | 0.0000444 | 0.33 | 422 |
| 19/20/21 | TP6 | MP | JHI-Hv50k-2016-92462 | 2 | 322598122 | 0.0000444 | 0.33 | 422 |
| 19/20/21 | TP6 | MP | JHI-Hv50k-2016-92557 | 2 | 342920995 | 0.0000444 | 0.33 | 422 |
| 19/20/21 | TP6 | MP | JHI-Hv50k-2016-92599 | 2 | 351064312 | 0.0000444 | 0.33 | 422 |
| 19/20/21 | TP6 | MP | JHI-Hv50k-2016-92887 | 2 | 361506229 | 0.0000444 | 0.33 | 422 |
| 19/20/21 | TP6 | MP | JHI-Hv50k-2016-92907 | 2 | 361788451 | 0.0000444 | 0.33 | 422 |
| 19/20/21 | TP6 | MP | JHI-Hv50k-2016-92985 | 2 | 365671468 | 0.0000444 | 0.33 | 422 |
| 19/20/21 | TP6 | MP | JHI-Hv50k-2016-93148 | 2 | 388134313 | 0.0000249 | 0.33 | 422 |
| 19/20/21 | TP6 | MP | JHI-Hv50k-2016-94189 | 2 | 423168003 | 0.0000444 | 0.33 | 422 |
| 19/20/21 | TP6 | MP | JHI-Hv50k-2016-94279 | 2 | 425643037 | 0.0000444 | 0.33 | 422 |
| 19/20/21 | TP6 | MP | JHI-Hv50k-2016-94875 | 2 | 444191228 | 0.0000444 | 0.33 | 422 |
| 19/20/21 | TP6 | MP | JHI-Hv50k-2016-94897 | 2 | 448196379 | 0.0000444 | 0.33 | 422 |
| 19/20/21 | TP6 | MP | JHI-Hv50k-2016-95108 | 2 | 461294870 | 0.0000444 | 0.33 | 422 |
| 19/20/21 | TP6 | MP | JHI-Hv50k-2016-95223 | 2 | 462141012 | 0.0000418 | 0.33 | 422 |
| 19/20/21 | TP6 | MP | JHI-Hv50k-2016-95334 | 2 | 463048352 | 0.0000444 | 0.33 | 422 |
| 19/20/21 | TP6 | MP | JHI-Hv50k-2016-95462 | 2 | 464675437 | 0.0000444 | 0.33 | 422 |
| 19/20/21 | TP6 | MP | JHI-Hv50k-2016-95515 | 2 | 465298467 | 0.0000418 | 0.33 | 422 |
| 19/20/21 | TP6 | MP | JHI-Hv50k-2016-95563 | 2 | 465651338 | 0.0000444 | 0.33 | 422 |
| 19/20/21 | TP6 | MP | JHI-Hv50k-2016-95877 | 2 | 469191114 | 0.0000444 | 0.33 | 422 |
| 19/20/21 | TP6 | MP | JHI-Hv50k-2016-96137 | 2 | 472013460 | 0.0000444 | 0.33 | 422 |
| 19/20/21 | TP6 | MP | JHI-Hv50k-2016-96228 | 2 | 473540782 | 0.0000444 | 0.33 | 422 |
| 19/20/21 | TP6 | MP | JHI-Hv50k-2016-96576 | 2 | 476627761 | 0.0000418 | 0.33 | 422 |
| 19/20/21 | TP6 | MP | JHI-Hv50k-2016-96611 | 2 | 476880621 | 0.0000444 | 0.33 | 422 |
| 19/20/21 | TP6 | MP | SCRI_RS_117951 | 2 | 360887764 | 0.0000444 | 0.33 | 422 |
| 19/20/21 | TP6 | MP | SCRI_RS_12448 | 2 | 360874294 | 0.0000444 | 0.33 | 422 |
| 19/20/21 | TP6 | MP | SCRI_RS_127347 | 2 | 464288056 | 0.0000444 | 0.33 | 422 |
| 19/20/21 | TP6 | MP | SCRI_RS_130072 | 2 | 362487172 | 0.0000444 | 0.33 | 422 |
| 19/20/21 | TP6 | MP | SCRI_RS_132839 | 2 | 469196911 | 0.0000444 | 0.33 | 422 |
| 19/20/21 | TP6 | MP | SCRI_RS_136740 | 2 | 469542752 | 0.0000444 | 0.33 | 422 |
| 19/20/21 | TP6 | MP | SCRI_RS_138697 | 2 | 461279676 | 0.0000444 | 0.33 | 422 |
| 19/20/21 | TP6 | MP | SCRI_RS_151921 | 2 | 226700347 | 0.0000444 | 0.33 | 422 |
| 19/20/21 | TP6 | MP | SCRI_RS_155067 | 2 | 363264632 | 0.0000444 | 0.33 | 422 |
| 19/20/21 | TP6 | MP | SCRI_RS_161169 | 2 | 432610472 | 0.0000444 | 0.33 | 422 |
| 19/20/21 | TP6 | MP | SCRI_RS_162917 | 2 | 462767055 | 0.0000444 | 0.33 | 422 |
| 19/20/21 | TP6 | MP | SCRI_RS_174318 | 2 | 361508091 | 0.0000444 | 0.33 | 422 |
| 19/20/21 | TP6 | MP | SCRI_RS_175300 | 2 | 361064839 | 0.0000444 | 0.33 | 422 |
| 19/20/21 | TP6 | MP | SCRI_RS_176173 | 2 | 269612591 | 0.0000417 | 0.33 | 422 |
| 19/20/21 | TP6 | MP | SCRI_RS_185513 | 2 | 230445616 | 0.0000444 | 0.33 | 422 |
| 19/20/21 | TP6 | MP | SCRI_RS_186769 | 2 | 471003862 | 0.0000481 | 0.33 | 422 |
| 19/20/21 | TP6 | MP | SCRI_RS_196026 | 2 | 473969305 | 0.000035 | 0.33 | 422 |
| 19/20/21 | TP6 | MP | SCRI_RS_197997 | 2 | 366354332 | 0.0000444 | 0.33 | 422 |
| 19/20/21 | TP6 | MP | SCRI_RS_208320 | 2 | 471739547 | 0.0000444 | 0.33 | 422 |
| 19/20/21 | TP6 | MP | SCRI_RS_208799 | 2 | 283783714 | 0.0000444 | 0.33 | 422 |
| 19/20/21 | TP6 | MP | SCRI_RS_220670 | 2 | 231381002 | 0.0000444 | 0.33 | 422 |
| 19/20/21 | TP6 | MP | SCRI_RS_231897 | 2 | 286185146 | 0.0000417 | 0.33 | 422 |
| 19/20/21 | TP6 | MP | SCRI_RS_232669 | 2 | 273436047 | 0.0000444 | 0.33 | 422 |
| 19/20/21 | TP6 | MP | SCRI_RS_237688 | 2 | 442045206 | 0.0000392 | 0.33 | 422 |
| 19/20/21 | TP6 | MP | SCRI_RS_83731 | 2 | 466243307 | 0.0000417 | 0.33 | 422 |
| 19/20/21 | TP6 | SP | JHI-Hv50k-2016-385463 | 6 | 42618343 | 0.000000154 | 0.29 | 422 |
| 19/20/21 | TP6 | SP | JHI-Hv50k-2016-49136 | 1 | 499170798 | 0.000032 | 0.15 | 422 |
| 19/20/21 | TP6 | SP | SCRI_RS_99344 | 5 | 594204409 | 2.02E-17 | 0.22 | 422 |
| 19/20/21 | TP6 | S/T | JHI-Hv50k-2016-195051 | 3 | 527696336 | 0.0000285 | 0.1 | 421 |
| 19/20/21 | TP6 | S/T | JHI-Hv50k-2016-365992 | 5 | 594326624 | 6.77E-08 | 0.31 | 421 |

Supplemental Table 7. Genetic correlations of the germination traits, germination rate (GI) at TP1 (6 days post PM), TP4 (48 days post PM), and TP6 (110 days post PM) to all malting quality traits collected for the 2020 and 2021 years (combined) and differing malting quality timepoints (TP) both overall *HvMKK3* alleles and on a per allele basis. Asterisks indicate significant increases in model likelihood (*** p < 0.001,** p < 0.01,* p < 0.05) and hence significance of correlation. Missing values or NA values indicate problems estimating standard errors of correlations or failed model convergence.

| **yearCor** | **Gtrait** | **TP** | **SD2Code** | **AA** | **BG** | **CCRU_Adj** | **CCRU_allMalt** | **DP** | **FAN** | **ME** | **MP** | **SP** | **S/T** |
| --- | --- | --- | --- | --- | --- | --- | --- | --- | --- | --- | --- | --- | --- |
| Combined | GE | TP1 | Overall |  | -0.547*** | 0.94*** | 0.119 | 0.199** | 0.831*** | 0.296*** | 0.271*** | 0.706*** | 0.723*** |
| 20/21 | GE | TP2 | Overall | 0.277*** | -0.539*** | 0.999*** | 0.48*** | 0.126* | 0.387*** | 0.344*** | 0.06 | 0.354*** | 0.394*** |
| 20/21 | GE | TP3 | Overall | 0.174** | -0.401*** | 0.692* | 0.229* | 0.109 | 0.179** | 0.175* | -0.097 | 0.119* | 0.245*** |
| Combined | GE | TP4 | Overall | 0.35*** | -0.526*** | 0.573*** | 0.308* | 0.125* | 0.333*** | 0.128 | -0.014 | 0.274*** | 0.395*** |
| 20/21 | GE | TP5 | Overall | 0.281* | -0.226 | 0.634 | -0.06 | 0.205 | 0.132 | 0.221 | -0.096 | 0.067 | 0.192 |
| Combined | GE | TP6 | Overall | 0.301** | -0.528*** | 0.768** | 0.109 | 0.317* | 0.302** | 0.177 | 0.154 | 0.251* | 0.24* |
| 20/21 | GE | TP7 | Overall | 0.317** | -0.339** | 0.999** | 0.399 | 0.2 | -0.13 | 0.245 | -0.19 | -0.2 | -0.025 |
| Combined | GE | TP1 | D | 0.468** | -0.495*** | 0.499 | -0.239 | 0.146 | 0.587*** | -0.198 | 0.241 | 0.459** | 0.298* |
| 20/21 | GE | TP2 | D | 0.367*** | -0.544*** |  | 0.232 | 0.196* | 0.509*** | 0.007 | 0.154 | 0.421** | 0.277* |
| 20/21 | GE | TP3 | D | 0.187 | -0.413*** |  | 0.081 | 0.218* | 0.365** | 0.1 | 0.176 | 0.363** | 0.203 |
| Combined | GE | TP4 | D | 0.517*** | -0.664*** | 0.77** | 0.275 | 0.217* | 0.459*** | -0.058 | 0.066 | 0.359** | 0.421** |
| 20/21 | GE | TP5 | D | 0.122 | -0.027 |  | -0.442 | 0.07 | 0.142 | 0.217 | 0.128 | 0.221 | 0.055 |
| Combined | GE | TP6 | D | 0.323* | -0.574*** | 0.84* | 0.048 | 0.352* | 0.451* | -0.192 | 0.259 | 0.329* | 0.157 |
| 20/21 | GE | TP7 | D | -0.188 | 0.213 | 0.362 | -0.141 | 0.411 |  | -0.019 | -0.195 | -0.658* | -0.606* |
| Combined | GE | TP1 | N | 0.221* | -0.243* | 0.727*** | 0.003 | 0.226* | 0.598*** | 0.257 | 0.311** | 0.564*** | 0.458*** |
| 20/21 | GE | TP2 | N | 0.045 | -0.311** | 0.419** | 0.545** | 0.208* | 0.072 | 0.753*** | 0.156 | 0.289** | 0.17 |
| 20/21 | GE | TP3 | N | 0.133 | -0.239 | 0.2 | 0.013 | 0.116 | -0.244 | 0.13 | -0.168 | -0.115 | 0.136 |
| Combined | GE | TP4 | N | 0.028 | -0.215 | 0.719*** | 0.233 | 0.136 | 0.094 | 0.361* | -0.013 | 0.239* | 0.373** |
| 20/21 | GE | TP5 | N | 0.269* | -0.211 | 0.55** | -0.032 | 0.366** | -0.023 | -0.03 | -0.041 | -0.128 | -0.047 |
| Combined | GE | TP6 | N | -0.018 | -0.02 | 0.156 | -0.54 | 0.089 | 0.095 | 0.322 | 0.206 | 0.158 | -0.037 |
| 20/21 | GE | TP7 | N | 0.188 | -0.342* | 0.297 | 0.55 | -0.053 | -0.274 | 0.39 | -0.193 | -0.407* | -0.156 |
| Combined | GE | TP1 | N* | 0.416** |  | 0.774** | 0.148 | 0.272 | 0.365 | 0.068 | 0.051 | 0.206 | 0.365 |
| 20/21 | GE | TP2 | N* | 0.217 |  | 0.782** | 0.47 | 0.269* | 0.244 | 0.24 |  | 0.159 | 0.056 |
| 20/21 | GE | TP3 | N* |  |  |  | 0.918 | 0.75*** | -0.642** | 0.303 |  | -0.171 | -0.275 |
| Combined | GE | TP4 | N* | -0.111 | -0.221 | 0.091 | -0.417 | 0.019 | -0.07 | 0.029 | -0.012 | -0.086 | -0.097 |
| 20/21 | GE | TP5 | N* | 0.865* |  |  |  | 0.523 | -0.371 | 0.706 |  | 0.297 | -0.17 |
| 20/21 | GE | TP7 | N* | 0.654 |  |  |  | 0.722** |  | -0.098 |  | -0.393 | -0.429 |
| Combined | GI | TP1 | Overall | 0.616*** | -0.517*** | 0.904*** | 0.119 | 0.162** | 0.858*** | 0.305*** | 0.26*** | 0.718*** | 0.751*** |
| 20/21 | GI | TP2 | Overall | 0.389*** | -0.591*** | 0.998 | 0.499*** | 0.105 | 0.611*** | 0.408*** | 0.119 | 0.521*** | 0.556*** |
| 20/21 | GI | TP3 | Overall | 0.369*** | -0.558*** | 0.999** | 0.428*** | 0.085 | 0.513*** | 0.293*** | -0.003 | 0.388*** | 0.504*** |
| Combined | GI | TP4 | Overall | 0.511*** | -0.532*** | 0.778*** | 0.239* | 0.163* | 0.644*** | 0.301*** | 0.081 | 0.517*** | 0.653*** |
| 20/21 | GI | TP5 | Overall | 0.347*** | -0.535*** | 0.999*** | 0.383* | 0.136 | 0.569*** | 0.433*** | 0.062 | 0.395*** | 0.447*** |
| Combined | GI | TP6 | Overall | 0.426*** | -0.532*** | 0.768*** | 0.391** | 0.194** | 0.549*** | 0.329*** | -0.002 | 0.374*** | 0.566*** |
| 20/21 | GI | TP7 | Overall | 0.417*** | -0.563*** | 0.999*** | 0.42* | 0.242** | 0.53*** | 0.538*** | 0.062 | 0.393*** | 0.446*** |
| Combined | GI | TP1 | D | 0.426** | -0.469*** | 0.447 | -0.232 | 0.112 | 0.592*** | -0.155 | 0.246 | 0.455** | 0.284* |
| 20/21 | GI | TP2 | D | 0.39*** | -0.539*** |  | 0.26 | 0.168 | 0.497*** | 0.059 | 0.115 | 0.384** | 0.28* |
| 20/21 | GI | TP3 | D | 0.322** | -0.511*** |  | 0.228 | 0.17 | 0.417** | 0.096 | 0.099 | 0.329* | 0.236 |
| Combined | GI | TP4 | D | 0.563*** | -0.61*** | 0.759*** | 0.147 | 0.211* | 0.632*** | 0.083 | 0.082 | 0.421*** | 0.442** |
| 20/21 | GI | TP5 | D | 0.461* | -0.57*** |  | 0.266 | 0.233 | 0.559** | 0.277 | 0.284 | 0.416* | 0.114 |
| Combined | GI | TP6 | D | 0.585*** | -0.681*** | 0.855*** | 0.426* | 0.345** | 0.411* | 0.157 | 0.015 | 0.245 | 0.315* |
| 20/21 | GI | TP7 | D | 0.331* | -0.512*** |  | 0.392 | 0.419** | 0.459* | 0.237 | 0.273 | 0.258 | -0.032 |
| Combined | GI | TP1 | N | 0.269* | -0.203 | 0.64*** | -0.068 | 0.191* | 0.563*** | 0.187 | 0.263** | 0.514*** | 0.45*** |
| 20/21 | GI | TP2 | N | 0.066 | -0.353** | 0.438*** | 0.402* | 0.211* | 0.241* | 0.622*** | 0.175 | 0.365*** | 0.258* |
| 20/21 | GI | TP3 | N | 0.159 | -0.367** | 0.419** | 0.172 | 0.234* | 0.136 | 0.262 | 0.065 | 0.204* | 0.203 |
| Combined | GI | TP4 | N | 0.129 | -0.297* | 0.776*** | 0.233 | 0.29** | 0.329** | 0.336* | 0.128 | 0.369*** | 0.482*** |
| 20/21 | GI | TP5 | N | 0.082 | -0.254* | 0.557*** | 0.218 | 0.316** | 0.205 | 0.394 | 0.105 | 0.156 | 0.074 |
| Combined | GI | TP6 | N | 0.128 | -0.266* | 0.676*** | 0.082 | 0.335** | 0.255* | 0.232 | 0.082 | 0.203* | 0.302* |
| 20/21 | GI | TP7 | N | 0.216 | -0.376* | 0.498** | 0.181 | 0.297* | 0.185 | 0.622* | -0.054 | 0.163 | 0.32* |
| Combined | GI | TP1 | N* | 0.201 | -0.589** | -0.039 | 0.37 | -0.099 | 0.856*** | -0.027 | -0.094 | 0.314 | 0.443* |
| 20/21 | GI | TP2 | N* | 0.322 |  |  |  | 0.002 | 0.524 | 0.2 |  | -0.052 | 0.007 |
| 20/21 | GI | TP3 | N* | 0.308 |  | -0.106 |  | -0.101 | 0.627* | -0.109 |  | 0.037 | 0.212 |
| Combined | GI | TP4 | N* | 0.14 | 0.2 | -0.401 | -0.305 | -0.136 | 0.456 | -0.02 | -0.153 | 0.046 | 0.189 |
| 20/21 | GI | TP5 | N* | -0.11 |  | -0.097 |  | -0.524* | 0.847* | 0.348 |  | -0.152 | 0.146 |
| Combined | GI | TP6 | N* | -0.214 | 0.21 | -0.351 |  | -0.412* | 0.596* | -0.053 | -0.448 | -0.262 | -0.029 |
| 20/21 | GI | TP7 | N* | 0.108 |  |  |  | -0.472 | 0.482 | 0.601 |  | -0.387 | -0.629 |

Supplemental table 8: Genetic (lower diagonal) and phenotypic correlations (upper diagonal) for the combined (19/20/21, treating malting timepoint as a replicate) dataset. Genetic correlations were derived from model 9, while phenotypic correlations were derived using the combined dataset derived BLUEs and the rcor() function within the Hmisc R package.

| MKK3 | Trait | **AA** | **BG** | **CCRU_Adj** | **CCRU_allMalt** | **DP** | **FAN** | **ME** | **MP** | **SP** | **ST** |
| --- | --- | --- | --- | --- | --- | --- | --- | --- | --- | --- | --- |
| All | AA | 1 | -0.36*** | 0.5*** | 0.06 | 0.45*** | 0.36*** | 0.06 | 0.1* | 0.35*** | 0.29*** |
|  | BG | -0.6*** | 1 | -0.46*** | -0.37*** | -0.19*** | -0.31*** | -0.14** | 0.11* | -0.19*** | -0.27*** |
|  | CCRU_Adj | 0.92*** | -1*** | 1 | 0.29*** | 0.58*** | 0.3*** | 0.24*** | 0.14** | 0.25*** | 0.2*** |
|  | CCRU_allMalt | 0.28 | -0.65** | 1*** | 1 | -0.1* | -0.14** | 0.38*** | -0.43*** | -0.25*** | 0.13** |
|  | DP | 0.4*** | -0.21* | 0.78*** | -0.37* | 1 | 0.13** | -0.15** | 0.46*** | 0.35*** | -0.08 |
|  | FAN | 0.59*** | -0.49*** | 0.54*** | 0.18 | 0.19* | 1 | 0.01 | 0.36*** | 0.69*** | 0.37*** |
|  | ME | 0.27** | -0.23* | 0.62** | 0.39* | -0.28** | 0.43*** | 1 | -0.29*** | -0.07 | 0.32*** |
|  | MP | 0.17* | 0.1 | 0.19 | -0.9*** | 0.59*** | 0.45*** | -0.34*** | 1 | 0.66*** | -0.2*** |
|  | SP | 0.57*** | -0.33*** | 0.57*** | -0.53*** | 0.51*** | 0.87*** | 0.04 | 0.76*** | 1 | 0.41*** |
|  | ST | 0.61*** | -0.6*** | 0.57*** | 0.56*** | 0.01 | 0.74*** | 0.51*** | -0.2** | 0.5*** | 1 |
| D | AA | 1 | -0.4*** | 0.47*** | 0.16 | 0.31*** | 0.3*** | 0.03 | -0.04 | 0.21* | 0.25** |
|  | BG | -0.49*** | 1 | -0.43*** | -0.47*** | -0.17 | -0.19* | -0.05 | 0.14 | -0.13 | -0.23** |
|  | CCRU_Adj | 1** | -1*** | 1 | 0.29*** | 0.54*** | 0.18* | 0.13 | 0.03 | 0.15 | 0.12 |
|  | CCRU_allMalt | 0.38 | -0.66** | 1 | 1 | -0.14 | -0.13 | 0.35*** | -0.49*** | -0.32*** | 0.13 |
|  | DP | 0.22 | -0.07 | 0.71* | -0.63** | 1 | 0 | -0.18* | 0.45*** | 0.34*** | -0.17 |
|  | FAN | 0.63** | -0.32* | 0.13 | -0.04 | 0.02 | 1 | -0.15 | 0.26** | 0.64*** | 0.31*** |
|  | ME | 0.27 | -0.26 | 0.02 | 0.32 | -0.29 | -0.25 | 1 | -0.3*** | -0.18* | 0.2* |
|  | MP | -0.17 | 0.19 | -0.35 | -0.92*** | 0.52*** | 0.38* | -0.41* | 1 | 0.67*** | -0.3*** |
|  | SP | 0.23 | -0.12 | 0.08 | -0.72** | 0.44** | 0.92*** | -0.27 | 0.76*** | 1 | 0.28** |
|  | ST | 0.49** | -0.42** | 0.76 | 0.48 | -0.2 | 0.66** | 0.22 | -0.51** | 0.19 | 1 |
| N | AA | 1 | -0.26*** | 0.4*** | 0.05 | 0.44*** | 0.05 | -0.01 | 0.1 | 0.13 | 0.04 |
|  | BG | -0.45* | 1 | -0.49*** | -0.27*** | -0.25*** | -0.3*** | -0.08 | 0.02 | -0.17* | -0.12 |
|  | CCRU_Adj | 0.55* | -0.87** | 1 | 0.33*** | 0.58*** | 0.25*** | 0.23*** | 0.26*** | 0.26*** | 0.11 |
|  | CCRU_allMalt | 0.42 | -0.47 | 1 | 1 | 0.01 | -0.2** | 0.37*** | -0.31*** | -0.21** | 0.09 |
|  | DP | 0.44** | -0.37* | 0.86*** | -0.11 | 1 | 0.12 | -0.18* | 0.47*** | 0.38*** | -0.09 |
|  | FAN | -0.08 | -0.24 | 0.43* | -0.07 | 0.14 | 1 | -0.09 | 0.48*** | 0.63*** | 0.13 |
|  | ME | 0.05 | 0.17 | 0.39 | 0.1 | -0.52** | 0.67** | 1 | -0.29*** | -0.13 | 0.27*** |
|  | MP | 0.01 | 0.07 | 0.52** | -0.84*** | 0.55*** | 0.62*** | -0.28 | 1 | 0.69*** | -0.2** |
|  | SP | 0.25* | -0.19 | 0.74*** | -0.7* | 0.61*** | 0.8*** | -0.08 | 0.84*** | 1 | 0.35*** |
|  | ST | 0.38* | -0.38* | 0.39 | 0.48 | 0.07 | 0.29 | 0.39 | -0.38** | 0.23 | 1 |
| N* | AA | 1 | -0.21* | 0.6*** | -0.04 | 0.76*** | 0.33** | -0.03 | 0.15 | 0.23* | 0.18 |
|  | BG | -0.22 | 1 | -0.41*** | -0.49*** | -0.17 | -0.15 | -0.24* | 0.34** | 0.11 | -0.27** |
|  | CCRU_Adj | 1*** | 0.42 | 1 | 0.3** | 0.61*** | 0.22* | 0.32** | -0.2 | -0.16 | 0.25* |
|  | CCRU_allMalt | -0.49 | -1* | 0.29 | 1 | -0.21* | -0.12 | 0.5*** | -0.65*** | -0.41*** | 0.32** |
|  | DP | 0.89*** | 0.04 | 1** | -0.37 | 1 | 0.24* | -0.13 | 0.36*** | 0.31** | -0.09 |
|  | FAN | 0.01 | 0 | -1 | 1 | -0.6 | 1 | -0.01 | 0.14 | 0.36*** | 0.23* |
|  | ME | -0.28 | -0.02 | 0.13 | 0.93* | -0.24 | 0.54 | 1 | -0.4*** | -0.3** | 0.43*** |
|  | MP | 0.68* | 0.23 | -0.19 | -1* | 0.54* | -0.09 | -0.67** | 1 | 0.74*** | -0.43*** |
|  | SP | 0.34 | -0.32 | -0.29 | -0.7 | 0.24 | 0.24 | -0.29 | 0.87* | 1 | -0.01 |
|  | ST | -0.2 | -0.55 | -0.71 | 0.96* | -0.43 | 0.37 | 0.49* | -0.39 | 0.11 | 1 |
